# Rationality, Irrationality and Escalating Behavior in Lowest Unique Bid Auctions

**DOI:** 10.1371/journal.pone.0029910

**Published:** 2012-01-18

**Authors:** Filippo Radicchi, Andrea Baronchelli, Luís A. N. Amaral

**Affiliations:** 1 Howard Hughes Medical Institute (HHMI), Northwestern University, Evanston, Illinois, United States of America; 2 Department of Chemical and Biological Engineering, Northwestern University, Evanston, Illinois, United States of America; 3 Departament d'Enginyeria Quimica, Universitat Rovira i Virgili, Tarragona, Catalunya, Spain; 4 Departament de Física i Enginyeria Nuclear, Universitat Politècnica de Catalunya, Campus Nord B4, Barcelona, Spain; 5 Northwestern Institute on Complex Systems (NICO), Northwestern University, Evanston, Illinois, United States of America; Universita' del Piemonte Orientale, Italy

## Abstract

Information technology has revolutionized the traditional structure of markets. The removal of geographical and time constraints has fostered the growth of online auction markets, which now include millions of economic agents worldwide and annual transaction volumes in the billions of dollars. Here, we analyze bid histories of a little studied type of online auctions – lowest unique bid auctions. Similarly to what has been reported for foraging animals searching for scarce food, we find that agents adopt Lévy flight search strategies in their exploration of “bid space”. The Lévy regime, which is characterized by a power-law decaying probability distribution of step lengths, holds over nearly three orders of magnitude. We develop a quantitative model for lowest unique bid online auctions that reveals that agents use nearly optimal bidding strategies. However, agents participating in these auctions do not optimize their financial gain. Indeed, as long as there are many auction participants, a rational profit optimizing agent would choose not to participate in these auction markets.

## Introduction

Animals searching for scarce food resources display movement patterns that can be statistically classified as Lévy flights [1]–[8]. Lévy flights [9] represent the best strategy that can be adopted by a searcher looking for a scarce resource in an unknown environment [10], and foraging animals seem therefore to have learned the best strategy for survival. Lévy flights describe also the movement patterns of humans in real space [11] and the variability of economic indices [12], but these observations do not correspond to search processes as in the case of foraging animals. Surprisingly, there is no indication of whether humans also use Lévy flight strategies when searching for scarce resources. Analyzing apparently unrelated data regarding online auctions, we address here this question and show that, when searching for scarce resources, humans explore the relevant space in the same class of strategies as foraging animals do.

**Figure 1:**
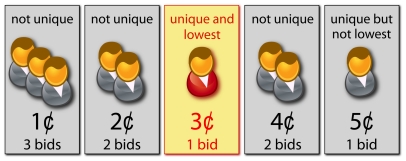
Unique bid auctions. Illustration of the rules of a lowest unique bid auction. At the end of the auction, the winner results to be the agent who has bid 3¢, which represents the lowest unique bid. All other bids are not unique apart from the one of 5¢, which is not the lowest one. In highest unique bid auctions the mechanism is reversed, and the winner is the agent making the highest unmatched bid.

Lowest unique bid auctions are a new generation of online markets [13]–[18]. Agents winning lowest unique bid auctions may purchase expensive goods for absurdly low prices; cars, boats and even houses can be bought for only hundreds of dollars. The idea of the auction is strikingly simple. A good, typically with a market value 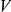 of at least a thousand dollars, is put up for auction. The auction duration is fixed *a priori*. A bid can be any amount from one cent to a pre-determined maximum value 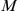, generally lower than one hundred dollars. Each time an agent makes a bid on a value 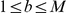, she pays a fee 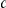, which ranges from one to ten dollars depending on the auction. During the bidding period, an agent knows only the status of her new bid, that is, whether it is winning or not. None of the agents knows on what values the other agents have bid until the end of the auction. When the bidding period expires, the agent who made the lowest unmatched bid can purchase the good for the value of the winning bid (see Fig. 1 for an illustration of the determination of the winning bid).

Lowest unique bid auction markets are competitive arenas. Each agent performs a search for a single target whose position changes from auction to auction, as it is determined by the bid history of the whole population of agents. Since the cost of each bid is as much as 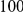 times larger than the natural unit of the bid, the number of bids that can be made by a single agent is limited and allows only a partial exploration of the bid space. Successful agents need to identify good strategies in order to maximize their winning chances and thus limit their risk.

Lowest unique bid auctions are just a particular variant of online pay-to-bid auctions, but other types of pay-to-bid auctions are regularly hosted on the web. For example, in highest unique bid auction the mechanism of lowest unique bid auction is inverted, and the winning bid is determined by the highest value closest to a pre-determined upper bound value. Since these auctions still involve a blind search of the winning value, highest unique bid auctions are equivalent to lowest unique bid auctions. Indeed, in this paper we analyze data taken from both types of auctions.

Other online pay-to-bid auctions, however, can be very different from lowest unique bid auctions. For example, the so-called penny auctions, which have acquired a great popularity in recent years, appear quite similar to but are not. As in the case of lowest unique bid auctions, the cost of the fee is at least 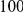 times larger of the bid increment, and as a consequence, the final value of the winning bid is much lower than the real value of the good up for auction. However, in penny auctions the value of the winning bid is publicly known and can only grow during the auction (i.e., the word “penny” is used because, in penny auctions, bid increments are equal to one cent). While escalation plays a very important role in penny auctions, in this type of auctions agents do not need to explore the bid space because the value of the winning bid is known. Penny auctions have been the focus of some theoretical and empirical studies [19]–[23].

## Results

We collected data from three distinct web sites hosting lowest unique bid auctions. We automatically downloaded and parsed the content of the tables reporting the bid history of closed auctions. These data sets contain all the information on individual auctions, including the details of each bid: its value, when it was made and who placed it. These data allow us to keep track of all the movements performed on bid space by a given agent bidding in a specific auction.

**Figure 2:**
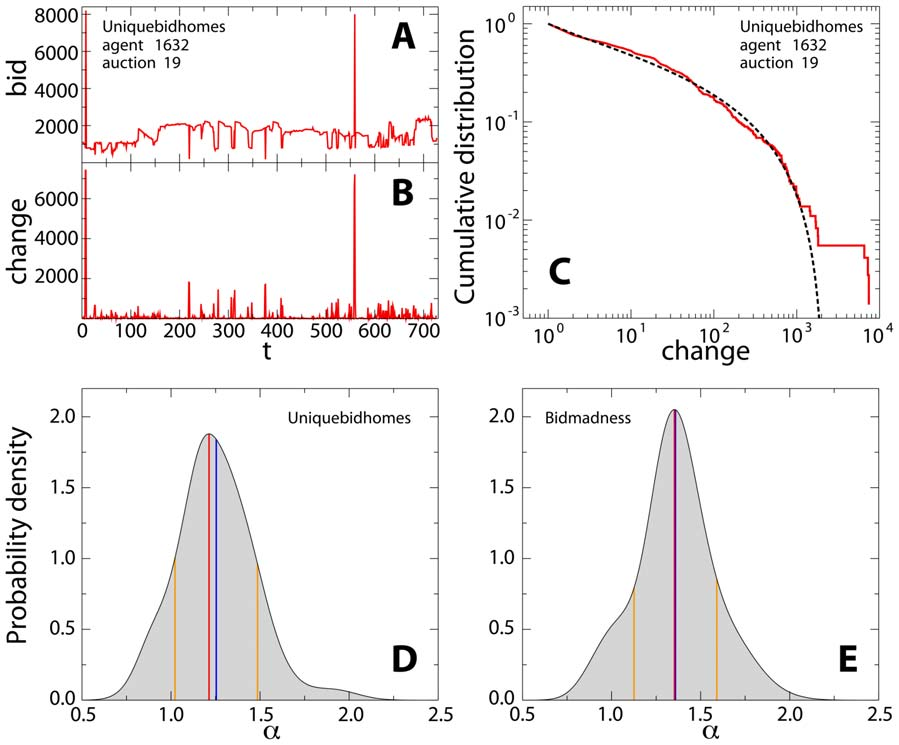
Individual activity. (**A**) Bid values explored by agent 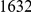 on auction 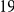 in the data set www.uniquebidhomes.com. Bids are sorted chronologically, and the figure reports the value 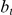 of the 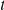-th bid. The unit of the bid amount is one hundredth of an Australian dollar. (**B**) Absolute value of the difference between two consecutive bids. The exploration of the bid space is characterized by a bursty behavior, where many small movements are occasionally followed by large jumps. (**C**) Cumulative distribution function of the change in bid value. The distribution is well fitted by a power-law, with decay exponent consistent with 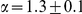 (dashed line). The agent therefore explores the bid space using a Lévy flight strategy. Notice that the curve bends down because of the finiteness of the bid space. (**D**) Probability density function of the Lévy-flight exponents adopted by agents in lowest unique bid auctions (www.uniquebidhomes.com). The blue line indicates the average value 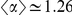 of the distribution, the red line identifies the mode 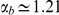 of the distribution, the orange lines bound the region within one standard deviation 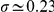 from the average. (**E**) Probability density function of the Lévy-flight exponents adopted by agents in highest unique bid auctions (www.bidmadness.com.au). In this case we find 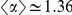, 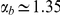 and 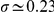.

**Figure 3:**
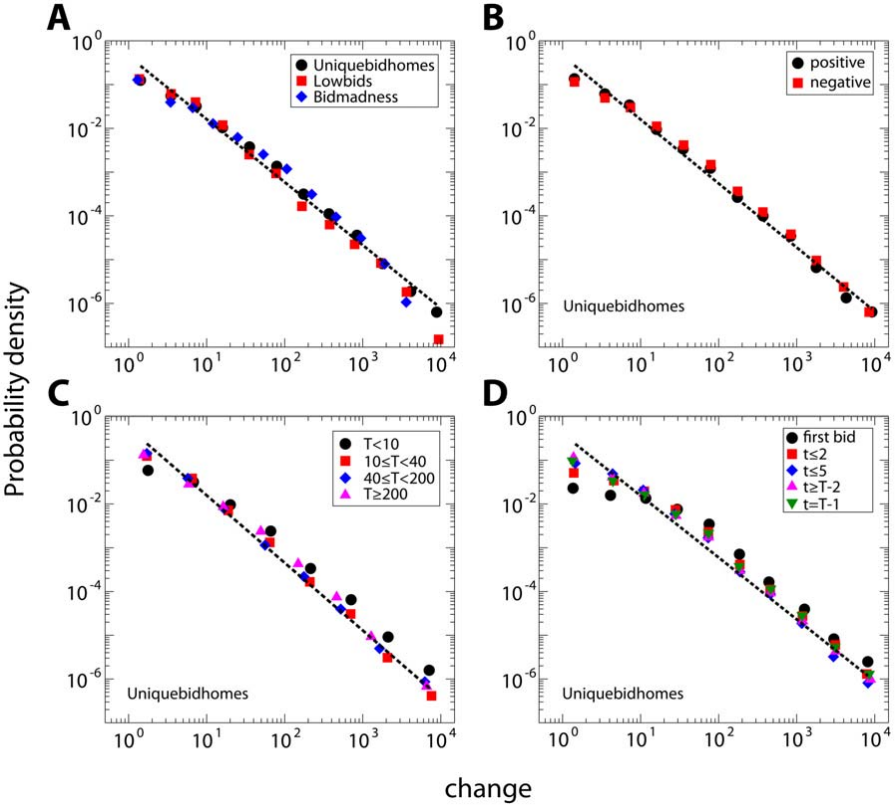
Bidding strategies of agents are Lévy flights. (**A**) Probability density function of the bid change for all agents in all auctions. We analyze data sets from three different web sites hosting auctions: www.uniquebidhomes.com (black circles), www.lowbids.com.au (red squares) and www.bidmadness.com.au (blue diamonds). (**B**) Probability density function of positive (black circles) and negative (red squares) bid changes. (**C**) Probability density function of the change amount for data aggregated over agents with different levels of activity (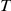 indicates the total number of bids made by an agent in a single auction). (**D**) Probability density function of the change amount at different stages of the auctions (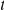 stands for order of the bid change in the bid history of an agent). In (A), (B) and (D) results have been obtained for lowest unique bid auctions (www.uniquebidhomes.com). All dashed lines stand for best power-law fits (least square) and all exponent values are consistent with 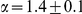. The unit of the bid value change amount is one hundredth of an Australian dollar.

We show in Figure 2A a typical exploration of the bid space performed by a single agent. The exploration of the bid space is bursty: consecutive bid values are generally close to each other, but from time to time the agent performs “long jumps” in bid space. We first compute the jump lengths (Fig. 2B) and estimate their probability distribution function (Fig. 2C). We find a strikingly robust power-law scaling consistent with the exploration of the bid space using a Lévy flight search strategy [9]. Note that here we use the notion of discrete Lévy flights. Time and space are in fact discrete, and the exploration of the bid space is modeled as a discrete time Markov chain [with transition probability defined in Eq. 8]. Our discrete model converges to a standard Lévy flight only in the continuum limit of space and time [24]. The power-law scaling can be observed both at the level of single agents (whenever the number of bids is sufficiently large for estimating the distribution; c.f. Figs. 2C and Supporting Information S1) and globally, by aggregating the length of the jumps made by all agents in all auctions (Figs. 3A and Supporting Information S1). The density distribution of the exponents calculated over single agents is peaked around a mean value 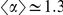 (Figs. 2D, 2E and Supporting Information S1), the same exponent value we estimate for the aggregated data. Significant variations around the average value are anyway present, and reflect the heterogeneity of the agent strategies. The density distributions of Figs. 2D and 2E are in fact calculated by considering different agents bidding in different auctions.

The power-law scaling and its measured exponent are very stable. Exponent estimates do not depend on the direction of the jumps (Figs. 3B and Supporting Information S1) or the level of activity of the agent (Figs. 3C and Supporting Information S1). Surprisingly, performing Lévy flights does not appear to be a learned strategy. Instead it appears to be an intrinsic feature of the mental search process: the jump lengths in the bid space follow the same power-law at any stage of the auction (Figs. 3D and Supporting Information S1).

Our results represent the strongest empirical evidence for the use of Lévy flight strategies in the search of scarce resources reported in literature up to now. Differently from previous studies where “two orders of magnitude of scaling can represent a luxury” [6], here the power-law decay can be clearly observed even over four orders of magnitude. It is unlikely, though, that adopting Lévy flight strategies is a deliberate choice of the agents, just as it is not likely that animals searching for food consciously follow a Lévy flight strategy. Nevertheless, the data demonstrate that the changes in bid value are statistically consistent with a power-law decaying distribution over several orders of magnitude (see and Supporting Information S1) [25]. Simple correlation measurements show also that the lengths of consecutive jumps are independent of each other (see and Supporting Information S1). We believe that the power-law is valid over such a broad regime because the space is not strictly physical. That is, movements of tens of thousands of cents can be performed for the same cost of those of only one cent. Agents thus explore the bid space in an effectively super-diffusive fashion, and steps are made with infinite velocity.

### Model

Next, we model the lowest unique bid auction process. Consider 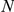 agents competing in a lowest unique bid auction. We model the successive bids of these agents as Lévy flight searches on bid space. Each agent moves in a bounded one-dimensional lattice with an *a priori* chosen exponent value, which may be regarded as the agent's strategy in the auction. In our formulation, every agent performs the same number 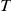 of bids and may return to already visited sites. At the beginning of the auction, every agent sits at the leftmost site on the lattice and then performs 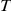 movements by changing, at each step, her actual position by an amount randomly drawn from a power-law distribution. If at stage 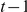 the agent with strategy 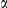 is sitting at position 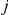, then at stage 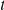 she jumps to position 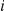 with probability proportional to 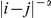. This model provides us with an independent way to determine the exponent values of the Lévy flights and offers a strikingly good statistical description of the data (Fig. 2B and Supporting Information S1).

We focus our attention on a generic agent bidding with strategy 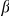 and on her chances to win auctions in which the rest of the population is bidding with strategy 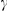. More complicated situations may in principle be studied with the same formalism.

### Single bid

Consider first the case in which agents make a single bid. The probability that a generic opponent, using bidding strategy 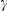, bids on value 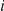 is
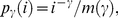
(1)with 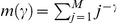 proper normalization constant. Here we consider the simple case in which all agents adopt the same bidding strategy 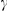. The probability of Eq. (1) can be anyway made more general by assuming that agents chose strategies from a density distribution 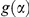 and calculating the probability of Eq. 1 as 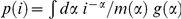. After all agents have bid, there will be 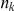 bids on the 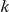-th bid value. Such variables clearly obey the constraint 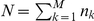. The probability to observe a particular configuration 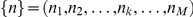 is given by
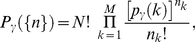
(2)which is a multinomial distribution with weights given by Eq. (1). In particular, the probability that only one bid (i.e., a unique bid) is made on value 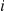 is
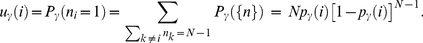
(3)


Focus now on the agent with bidding strategy 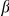. The probability that, making a bid on value 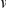, she makes a lowest unique bid can be calculated exactly by summing the multinomial distribution of Eq. (2) over all configurations for which there are no bids on the value 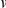 and there is not a unique bid on a value smaller than 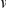, and finally multiplying this factor by the probability that the agent with bidding strategy 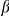 bids on the value 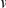. Such exact calculation is however unfeasible due to the extremely high number of possible combinations, and therefore we approximate the probability that, making a bid on value 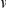, the agent with bidding strategy 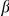 makes a lowest unique bid as
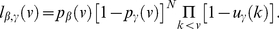
(4)The r.h.s. of Eq. (4) is the product of three terms: 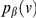 is the probability that the agent bids on value 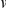; 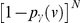 is the probability that none of the opponents have bid on value 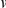; 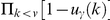 is the probability that none of the bid values smaller than 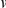 are occupied by a single bid made by one of the opponents. In spite of the fact that Eq. (4) is just an approximation of the real 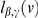, the approximation can be considered good because able to reproduce the results obtained from the direct simulation of the process (see the section Results). Moreover in the simplest case in which 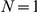, it correctly reduces to the exact value 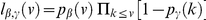.

Finally, the probability that the agent with bidding strategy 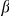 wins the auction is
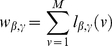
(5)and, on average, the value of her winning bid is
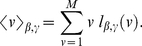
(6)


### Repeated auctions

Imagine now to repeat the same auction 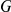 independent times. The probability that the agent bidding with strategy 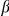 wins 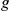 times out of 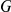 total auctions is given by a binomial distribution
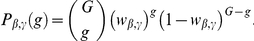
If the agent with bidding strategy 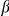 wins 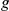 auctions, the sum of her winning bids is a random variable 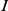 whose probability is determined by
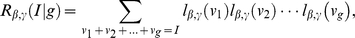
where the sum runs over the integer indices 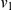, 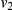, …, 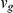 with the constraint that their sum should equal 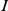. Excluding bidding costs, the average return of the agent in 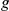 victories is
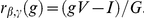
In general, the probability that the sum of the winning bids is equal to 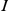 in an arbitrary number of auctions won by the player with bidding strategy 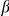 can be calculated as
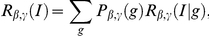
and a similar expression can be derived for the distribution of 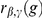. However, we are interested in the case in which the number of auctions diverges (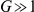). In this limit, we can approximate the number of victories with its average 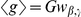 as well as the sum of the winning bids as 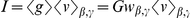. The return of the agent with bidding strategy 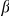 is therefore
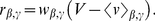
(7)For 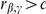, the agent has a positive return for participating in the auction, whereas, for 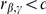, her return is negative.

### Multiple bids

Given a generic agent with bidding strategy 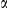, her first bid is placed on value 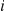 with probability 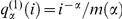. For the subsequent bids, we need to define a transition matrix 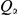, whose generic element 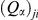 gives the probability that the agent bids on value 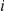 when her previous bid has been made on value 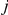. In our model, we have
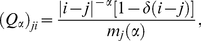
(8)for all 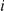 and 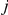 in the interval 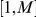. 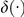 is the Kronecker delta, equal to one if its argument is equal to zero, and equal to zero otherwise. The normalization constant 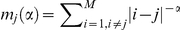 ensures the proper definition of the transition matrix. The matrix 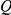 describes a random walker performing uncorrelated Lévy flights with exponent 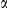. Notice that the agent has no memory of her previous bid values and therefore she may place more than a bid on the same value. At the generic step 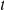, the probability that the agent with bidding strategy 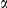 bids on the value 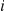 is
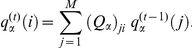
The probability that this agent has bid, during her 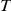 bids, on value 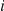 is then
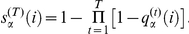
The term 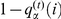 counts the probability that the agent has not bid on value 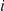 at stage 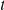. The probability that the agent has not bid on value 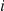 at any stage is therefore the product of this single step probabilities. Finally, the probability that the agent has bid on value 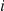 at least once is calculated as the probability to have bid on value 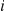 an arbitrary number of times minus the probability to have never bid on value 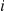.

Now go back to the situation in which an agent with bidding strategy 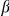 is opposed to a population of 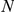 agents with bidding strategy 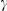. The probability that the agent with bidding strategy 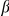 has bid, in 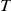 steps, at least once on value 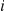 is 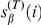. The probability that one of the 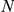 opponents, bidding with strategy 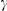, makes a unique bid on value 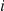 is given by
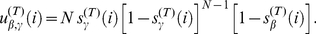
(9)

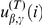
 is the product of two terms: 
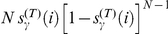
 is the probability that a bid on value 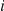 is unmatched by any of the other 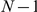 opponents, while 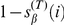 is the probability that also the agent, with bidding strategy 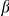, does not bid on value 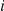. The probability that the agent with strategy 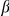 wins the auction with a bid on value 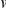 is
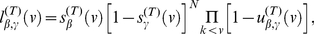
(10)respectively standing for the product of the probabilities that: she bids on value 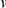; none of the other agents bids on value 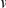; none of the bids with value smaller than 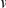 is unique. Eqs. (9) and (10) represent the generalization of Eqs. (3) and (4), respectively. In Eq. (10) we made the same type of approximation as the one used for writing Eq. (4). The probability 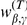 that the agent with bidding strategy 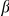 wins the auction and the average value 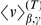 of her winning bids can be respectively calculated using Eqs. (5) and (6). Finally, excluding bidding costs, the return 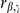 of the agent with strategy 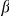 over an infinite number of auctions is again given by Eq. (7). For 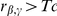, the agent has a positive return for participating in the auction, whereas, for 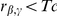, her return is negative.

### Model predictions

**Figure 4:**
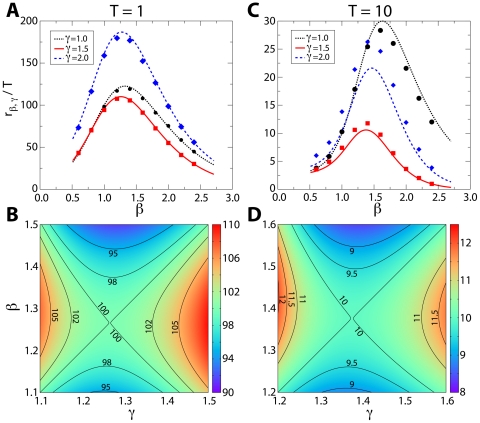
Model predictions. Economic return 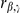 [Eq. (7)], divided by the number of bids 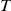, of an agent bidding with strategy 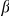 when competing, in a lowest unique bid auction with upper-bound 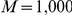 and for a good of value 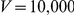, against 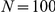 opponents bidding with strategy 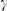. Unless specified, the quantity 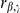 reported in this plots is computed by numerically solving the equations of the model. (**A**) Case where each agent performs a single bid in the auction, for three values of 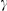. Theoretical predictions (lines) are compared with the results of numerical simulations (symbols). In each simulation of the auction, we randomly extracted 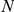 bid values 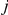 with probability proportional to 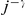, and a single bid value 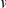 with probability proportional to 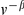. For a given set of parameters, we repeated the same simulation 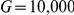 times, and calculate the number of times 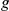 in which the bid value extracted from the power-law distribution with exponent 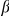 was the winning bid, and the sum 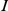 of these winning bid values. The economic return has been finally calculated as 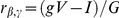. (**B**) Exploration of parameter space reveals the existence of a saddle point at 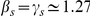. (**C**) Case where each agent performs 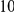 bids in the auction, for three values of 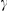. Numerical simulations have been carried out as in the former case, but considering agents moving in the bid space according to Eq. (8). (**D**) Exploration of parameter space reveals the existence of a saddle point at 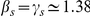.

We show in Fig. 4 the results obtained with our analytical model. The presence of a saddle point at 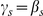 indicates that 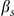 is an optimal strategy or Nash equilibrium [26]–[28]. When the opponents do not bid rationally (i.e., 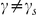), it is more convenient to use a strategy 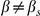. On the other hand, when the other agents bid rationally (i.e., 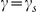), there is no better strategy than 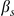. The value of 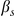 depends on the parameters 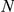 and 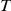, but for realistic choices (see and Supporting Information S1 and Fig. 4), 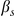 is in the range 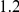 to 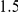, the same range of the exponent values we estimated from the data. Thus, despite its simplicity, our model captures the main features of the real auctions. Performing Lévy flights with small exponents (ballistic motion) yields unique bids that are unlikely to be the lowest. On the other hand, performing preferentially short jumps (high exponents, diffusive motion) guarantees to always bid on small values which are unlikely to be unique. Intermediate values of the exponent (super-diffusive motion) represent a compromise between staying low and being unique, and therefore lead to maximal winning chances. These considerations are valid only for finite values of 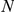 and 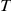, which is the realistic case. Because the available positions in the lattice are finite, when either 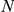 or 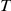 grow, the probability to observe a unique bid progressively approaches zero [29]. Notice that at the saddle point 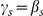, all 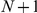 agents are using the same bidding strategy and therefore they all have the same chances to win the auction. In particular, the probability that a generic agent wins the auction is 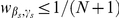, where the inequality may arise because a unique and lowest bid may not exist.

The value of the exponent, corresponding to the optimal Lévy flight strategy in lowest unique bid auctions, is distinct from the one found in the case of purely random searches [10], and empirically observed in the movement patterns of foraging animals [1]–[8]. The quantitative difference arises, we believe, as a consequence of the anisotropy of the bid space (low values are favored), the role of competition, and, more importantly, the fact that the target is not “static” but moving according to the actions of the whole population of agents.

## Discussion

**Figure 5:**
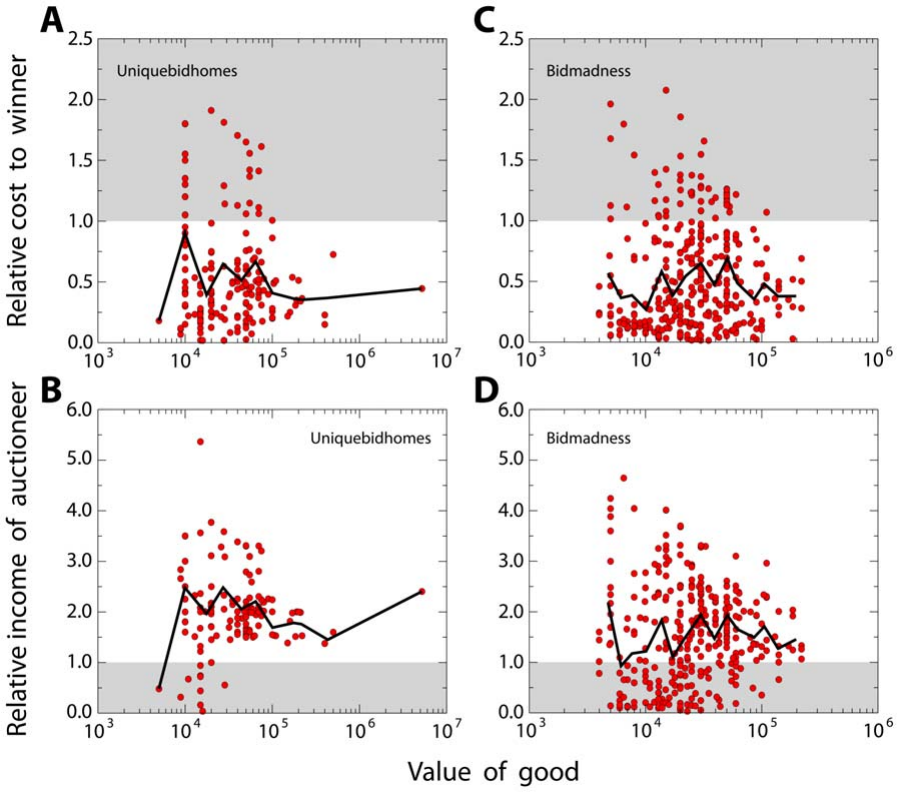
Economic return of agents. Auction winners tend to pay half of the value of the good, while auctioneers tend to earn twice the value of the good. We consider two different data sets, one regarding lowest unique bid auctions [www.uniquebidhomes.com, (A) and (C)] and the other highest unique bid auctions [www.bidmadness.com.au, (B) and (D)]. The unit is one hundredth of an Australian dollar. (**A**) Relation between the relative cost 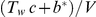 to the winner of an auction and her income 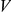. 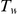 indicates the number of bids made by the winner of the auction, 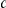 is the cost of the fee, 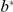 indicates the value of the winning bid, and 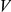 is the value of the good put up for auction. Each point represents an auction. The gray area corresponds to the region of negative return for winners of the auctions. The black line indicates the average value of the relative cost to the winners. Auctions are, on average, very profitable for the agent winning the auction, but the probability of a specific agent winning the auction is very low. Data refer to the data set www.uniquebidhomes.com. (**B**) Relative income 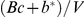 of the auctioneers as a function of their investment 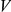. 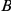 is the number of bids placed by all agents. The gray area denotes the region of negative return for the auctioneers. The black line indicates the average value of the relative profit of the auctioneers. On average, organizing auctions is very profitable. Data refer to the data set www.uniquebidhomes.com. (**C**) Relation between the relative cost to the winner of an auction and her income for the data set www.bidmadness.com.au. (**D**) Relative income of the auctioneers as a function of their investment for the data set www.bidmadness.com.au.

In lowest unique bid auctions, agents have the possibility to win goods of high value for impossibly low prices (Figs. 5A and 5C), However, these all-pay auction markets are designed to be very profitable for the auctioneers [30]–[33], who, on average, double their investment (Figs. 5B, 5D and Supporting Information S1). For auctioneers, the profitability of lowest unique bid auctions is in fact guaranteed by the validity of the inequality 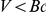, where 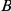 stands for the total number of bids and equals 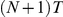 in our model. Under this constraint however, the payoff of a generic agent in a perfectly rational population is always negative since
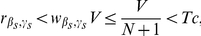
and there is no expected economic gain to be obtained for participating as a bidder in the auction markets. The rationality of the economic agents in adopting optimal strategies seems, therefore, in contrast with the ultimate irrationality that induces agents to take part in these auction markets.

Competitive irrationality, based on rational choices, has been investigated in economic theories [34]–[37], such as the dollar auction game [38]. The decision to participate or not participate in lowest unique bid auctions presents a paradox for potential bidders. If the number of agents participating in the auction is not too high, then the auction would bring a positive economic return to the agents, but not to the auctioneers. For example, in the case in which only one bidder participates in the auction, this bidder would have the maximal economic return by placing a single bid on the lowest value allowed. But by this token, every agent will feel that participating is profitable as long as not many other agents have bid yet. However, no agent can know how many other agents will actually bid on the good.

Our results raise a number of important research questions. First, which brain regions are responsible for implementing the search strategies used by agents? Since agents use similar search strategies to bees or birds, it is likely that there is no frontal cortex involvement. Using neuroimaging techniques such as fMRI it should be possible to answer this question. Second, does the economic paradox that the agents face reveal itself in brain activity patterns? Specifically, do some of the changes in brain activity observed for preference reversal [39], [40] occur also in this case? Additionally, our results suggest that controlled lowest unique bid auction markets would offer the possibility to run large-scale experiments at relatively low cost [41]. These experiments could be used for monitoring the behavior of agents in auction markets with tunable optimal search strategies, and see if (and how fast) agents are able to adapt their behavior to optimality.

## Materials and Methods

Data have been collected from three publicly accessible web sites: www.uniquebidhomes.com, www.lowbids.com.au and www.bidmadness.com.au. Also, we make available a version of these data at the web page filrad.homelinux.org/resources.

## Supporting Information

Supporting Information S1 “Lowest Unique Bid” and “Highest Unique Bid” Auctions.
(PDF)
