# Supplementary material for: Rationality, Irrationality and Escalating Behavior in Lowest Unique Bid Auctions
Source: PLoS One. 2012 Jan 18;7(1):e29910. doi: 10.1371/journal.pone.0029910 (PMC3261157; doi:10.1371/journal.pone.0029910)
Supplement: Supporting Information S1 — “Lowest Unique Bid” and “Highest Unique Bid” Auctions. (PDF) [file pone.0029910.s001.pdf]

Supporting information: "Rationality, irrationality and escalating behavior in online auctions"

Filippo Radicchi,[1] Andrea Baronchelli,[2] Luis A. N. Amaral[1,3,4,*]
[1]Howard Hughes Medical Institute (HHMI), Northwestern University,
Evanston, Illinois 60208 USA
[2]Departament de Física i Enginyeria Nuclear, Universitat Politècnica de Catalunya,
Campus Nord B4, 08034 Barcelona, Spain
[3]Department of Chemical and Biological Engineering, Northwestern University,
Evanston, Illinois 60208 USA
[4]Northwestern Institute on Complex Systems (NICO), Northwestern University,
Evanston, Illinois 60208 USA
[*]To whom correspondence should be addressed; E-mail: amaral@northwestern.edu.

S 1 "Lowest Unique Bid" and "Highest Unique Bid" Auctions

S 1.1 Description of the auction

Lowest Unique Bid (LUB) auctions are special on-line auctions which have reached a considerable success during last years. Their peculiarity consists in the fact that they are reverse auctions: rather than the bidder with the highest bid (as in the case of traditional auctions), the winner is the person who makes the LUB (see Figure 1a in the main text). The rules of a LUB auction are very simple. At the beginning of each auction, the auctioneers put up for auction a good of value V. After the beginning of the auction and for a certain period of time (in general of order of weeks), agents participate to the auction by making bids. The natural unit of the auction is one hundredth of dollars, euros, etc (i.e., the currency depends on the country where

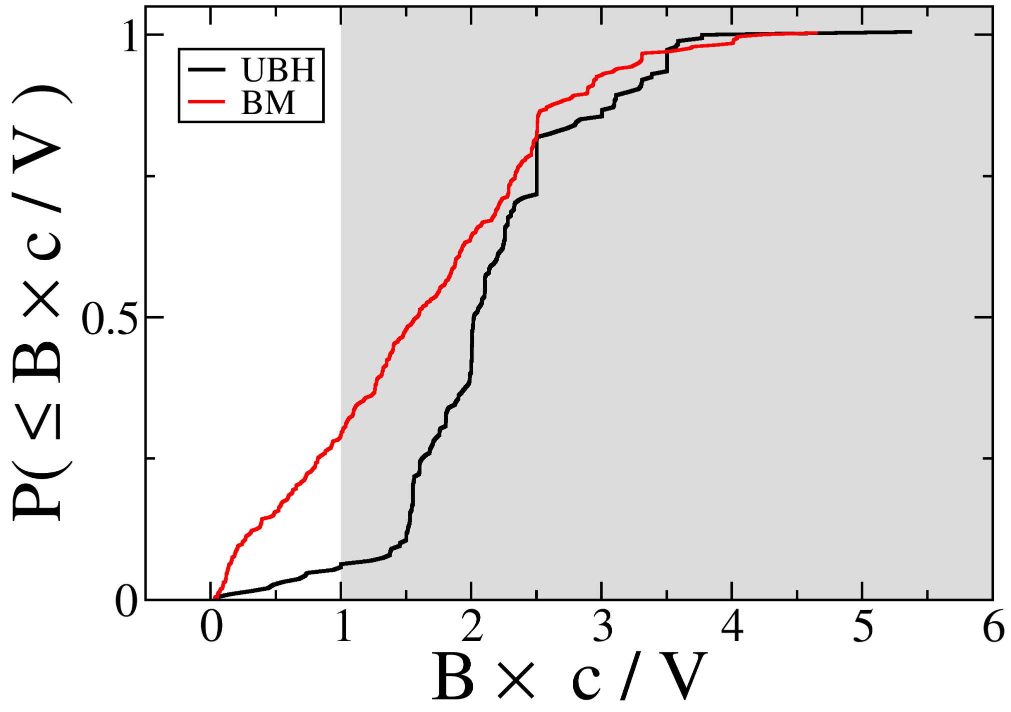

Figure S1: Cumulative distribution of the relative return $(B \times c)/V$ for the auctioneers. For `www.uniquebidhomes.com` data set (black line) the 95% of the auctions are in the gray region of positive return, where the relative return $(T \times c)/V > 1$. On average, the relative return of the auctioneers is 2.2. For `www.bidmadness.com.au` data set (red line) the 72% of the auctions have produced a positive relative return and on average the relative return is 1.6.

the auction is hosted). Bids may be any amount (in cents) between one cent and a maximal bid amount M (generally lower than ten hundreds of cents). Sometimes, the value of M is not fixed, but the bid space is anyway naturally bounded since none of the agents wants to bid more than V. The value of V depends on the auction, but generally its order of magnitude is of thousands of hundreds of cents. Making a bid costs a fee c (typically from one hundred to ten hundreds cents). After each bid b, the agent receives an automatic message, from the web site hosting the auction, saying whether that bid was the winning bid (i.e., the bid is the LUB) or not. The

agent is constantly informed about the status of her bids (i.e., whether one of them becomes the LUB or is not longer the LUB). However, each agent knows only what she has bid, without any information on which values the other agents have bid. In general, there is no restriction for the number of bids that the same agent may place. When the time dedicated to the auction expires, the winner is the agent who made the LUB and can therefore purchase the good for the value of her winning bid. If at the close of the auction a single lowest unique bid does not exist, the successful bid becomes the lowest one made by only two agents and the winner is the one who has bid first on such value. In the case in which also a bid made by only two agents does not exists, then the winning bid becomes the lowest one made by only three agents and so on. Again in these situations, the winner is the agent who has first placed a bid on the winning value. In our data sets however, we always observe that the winning bid is an unmatched bid.
There are several slight variations of this kind of auctions. Very often, the end of the auction is not determined by an expiration time, but by a minimum required number of bids, *a priori* fixed by the auctioneers. In other variations called Highest Unique Bid (HUB) auctions, the winning bid is the unique one closest to M.
Independently on the type of auctions, this kind of auctions are particularly profitable for both the auctioneers and the winners of the auctions. Figure 5 of the main text and Figure S1 clearly show that there are only few exceptions in which auctioneers or winners have lost money, but in the majority of the auctions their returns are positive.

Data set	Tot. Auctions	Tot. Agents	Tot. Bids	$\langle M \rangle$	$\langle c \rangle$	$\langle N \rangle$	$\langle B \rangle$
UBH	189	3 740	55 041	362	437	50	6
LB	55	445	3 740	1 284	478	13	6
BM	336	3 719	127 275	504	174	40	14

Table S1: Summary table of the data sets analyzed in this paper. We report, from left to right, the name of the data set, the total number of auctions, the total number of different agents, the total number of bids, the average value of the maximal bid value, the average amount of the fee, the average number of agents involved in an auction and the average number of bids made by a single agent in a single auction. The unit of the bid values is one hundredth of an Australian dollar.

S 1.2 Description of the data sets

We collected data from the web sites `www.uniquebidhomes.com` (UBH),

`www.lowbids.com.au` (LB) both hosting LUB auctions and

from `www.bidmadness.com.au` (BM) organizing HUB auctions. Data regard all auctions organized during 2007, 2008, 2009 and part of 2010 by these web sites. We collected detailed information concerning the auctions: the value of the goods, the cost of the fee, the maximum bid amount, the duration of the auction or eventually the required number of bids. We report in Table S1 some of these quantities calculated for our data sets. We were able also to keep track, for all data sets, of each single bid, getting information about its value, the time when it was made and the agent who made it. Data sets were anonymised and can be downloaded at the page `filrad.homelinux.org`. In the following, we focus our analysis mainly on the data sets UBH and BM since, given their size, allow to perform much better statistics.

S 1.3 Analysis

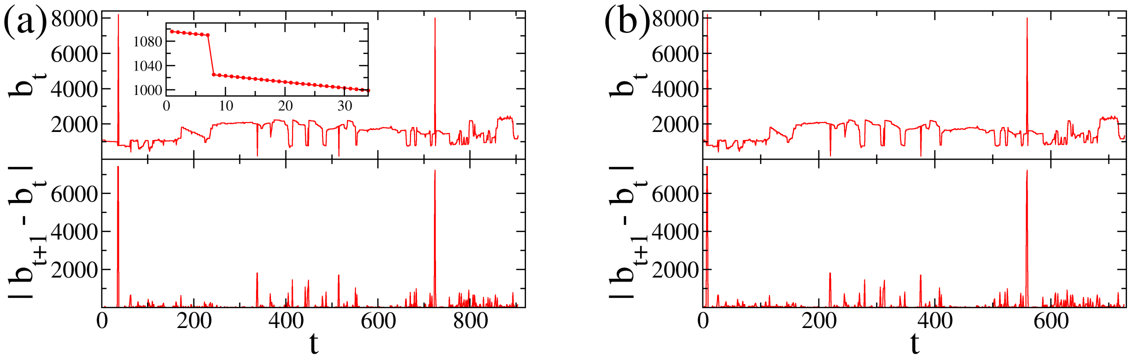

Figure S2: UBH data set. (a) In the top panel, the time series of bid values performed by agent $u = 1632$ in auction $a = 19$ is shown. In particular we zoom into a region where "abnormal" movements are present. In the bottom panel, we plot the time series of the length of her jumps $d_t = |b_{t+1} - b_t|$. (b) Same plots as those appearing in panel a, but for the cleaned version of the time series. The indices a and u refer to the anonymised version of the UBH data set.

Fixed an auction a and an agent u, we consider the temporal series of her bids, whose total number is denoted by $T_{a,u}$. This series is basically a list of integers $b_1, b_2, \ldots, b_T$, where we have suppressed the indices a and u for shortness of notation. Their value is defined over the interval $[1, M]$. All bs are different each other since no agent bids on a certain value more than once. A typical example of these time series is reported in the top panel of Figure S2a. We calculate the gap or difference between subsequent bid values and indicate it with $d_t = |b_{t+1} - b_t|$. Given a list of T bid values we can in fact extract a list of $T - 1$ differences between consecutive bids. In the bottom panel of Figure S2a, we plot the time series d_t for the same agent whose bid time series is plotted in the upper panel.

S 1.4 Cleaning the data sets

Generally, more "professional" agents perform random searches followed by systematic coverages of intervals. A typical example is shown in the inset of the upper panel of Figure S2a. Here a zoom of the time series appearing in the main plot is reported. Systematic coverages are performed by selecting a range of bid values and then placing a bid on each single value in that interval. This is an opportunity offered by the web site hosting auctions. Each bid in this case is characterized by the same time stamp. Such occurrence is likely for agents who make a significant number of bids, but becomes less relevant for agents who invest relatively small amount of money. We cleaned data by removing all pieces of the time series corresponding to this "abnormal" behavior. It should be remarked that the gaps between consecutive bids which can be measured in these regions are in the majority of the cases equal to one. Including these regions will influence only gaps equal to unity by overestimating their presence. We decided to remove such systematic coverages in order to focus our attention only on "normal" bidding strategies. The result, after the cleaning procedure of time series shown in Figure S2a, is reported in the upper panel of Figure S2b. The gaps between consecutive bids in the cleaned time series are reported in the bottom panel of Figure S2b.

As additional information, in Figure S3, we measure the number of agents $N(\rho)$ performing a ratio ρ of bids made using a normal strategy (i.e., after removing systematic coverages) and the total number of bids placed.

In the following analysis, we consider only cleaned time series, where systematic coverages have been deleted.

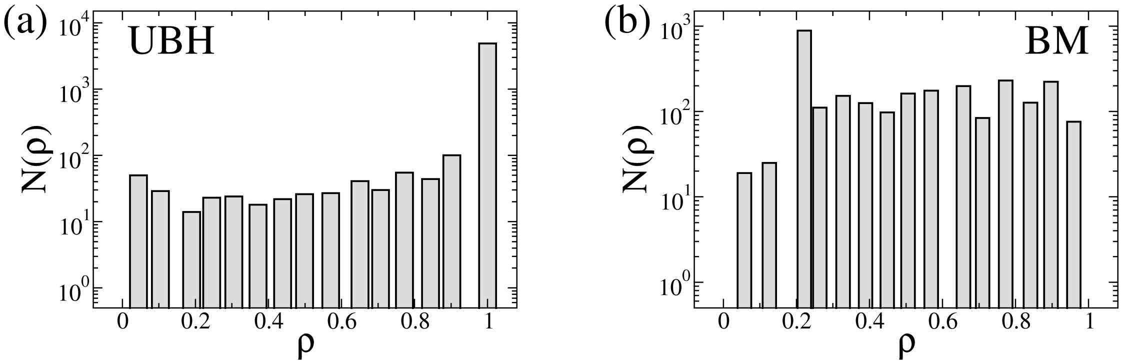

Figure S3: Number of agents $N\left(\rho\right)$ performing "normal" search strategies at rate ρ.

S 1.5 Statistics of the length of the jumps

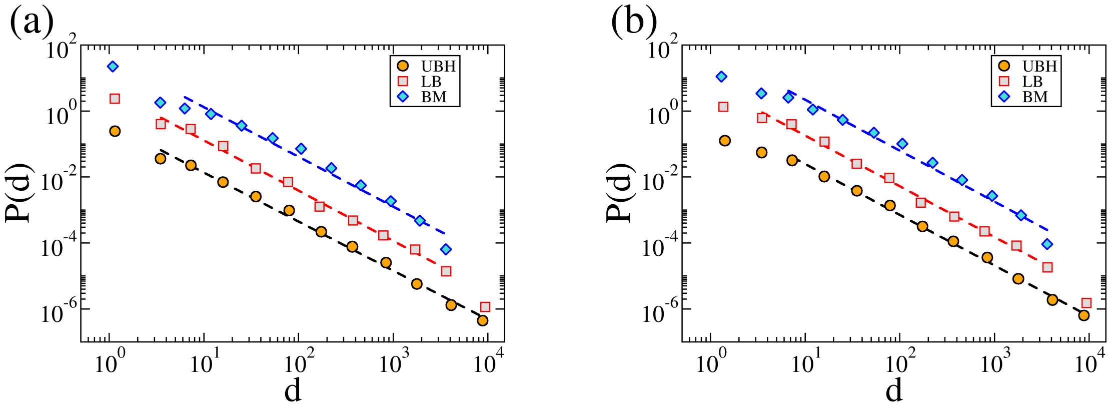

Figure S4: (a) Probability distribution function $P(d)$ calculated over all agents and auctions in the data sets UBH (orange circles), LB (gray squares) and BM (turquoise diamonds). Dashed lines stand for best power-law fits (least square). We find $\alpha = 1.54(2)$ [black], $\alpha = 1.54(3)$ [red] and $\alpha = 1.54(5)$ [blue]. (b) Same as in panel a, but for cleaned time series. Dashed lines have slopes $\alpha = 1.49(2)$ [black], $\alpha = 1.52(2)$ [red] and $\alpha = 1.51(5)$ [blue]. The data of this figure also reported in Figure 3A of the main text. Curves calculated for LB and BM data sets have been vertically shifted for clarity.

We measure the probability distribution function (pdf) $P(d)$ of the difference between subsequent bids made by single agents in single auctions. Global (i.e., aggregated over all agents and auctions) pdfs of both data sets are plotted in Figure S4. For agents with a sufficient number of bids in the same auction, we also compute individual pdfs. Some examples are reported in the various panels of Figures S5, S6 and S7 for UBH data set and in Figures S8, S9 and S10 for BM data set. In each panel of these figures, we explicitly indicate the id of the auction a and the id of the agent u as they appear in our anonymised version of the data sets. In all cases, we find a behavior compatible with

$$P(d) \sim d^{-\alpha} \quad . \tag{S1}$$

The search strategy adopted by agents is therefore given by Lévy flights with characteristic

exponent α. The best fits with power-laws are plotted in Figures S5, S6, S7, S8, S9 and S10 with black dashed lines and the value of α, plus the associated error, corresponding to the best fit is reported at the top of each panel.

In order to calculate a pdf, we divide the range of possible values of d in bins equally spaced on the logarithmic scale. We then drawn the pdf by associating to each bin the number of ds falling in that bin divided by the number of integers that could enter in the bin (this because d can assume only integer values). Everything is then normalized by simply dividing by the total number of points. We compute the best power-law exponent by performing a linear least square fit in double logarithmic scale. Despite the binning procedure may introduce a certain amount of arbitrariness in the evaluation of the pdfs, we checked the consistency of our results by varying the number of bins. Moreover, we additionally make use of a different fit method (maximum likelihood) about which we will discuss later.

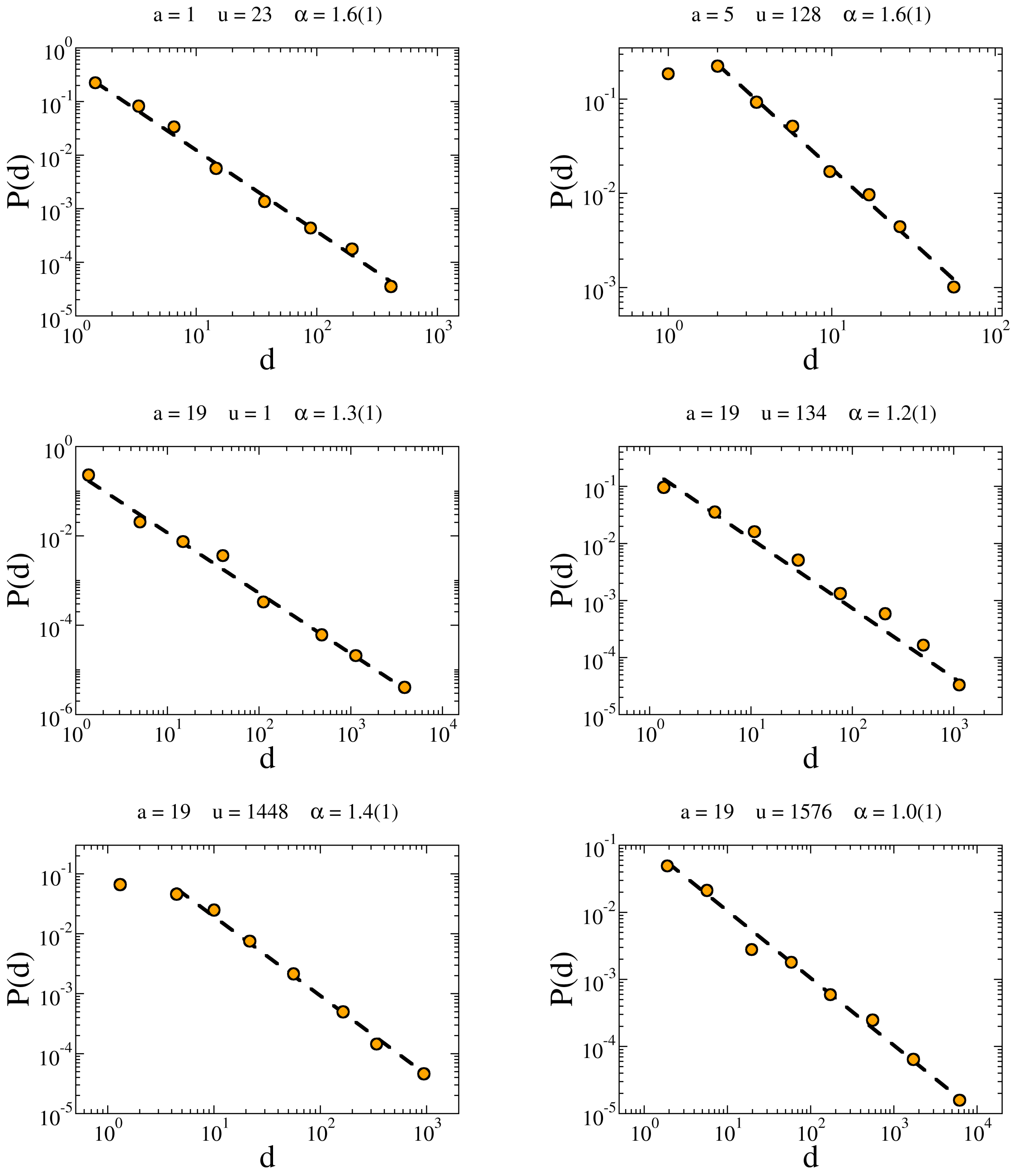

Figure S5: UBH data set. Probability distribution function $P(d)$ measured for agent u in auction a. We show several $P(d)$s for different pairs u and a. Dashed lines denote the best power-law fit (least square) $P(d) \sim d^{-\alpha}$ obtained for the data.

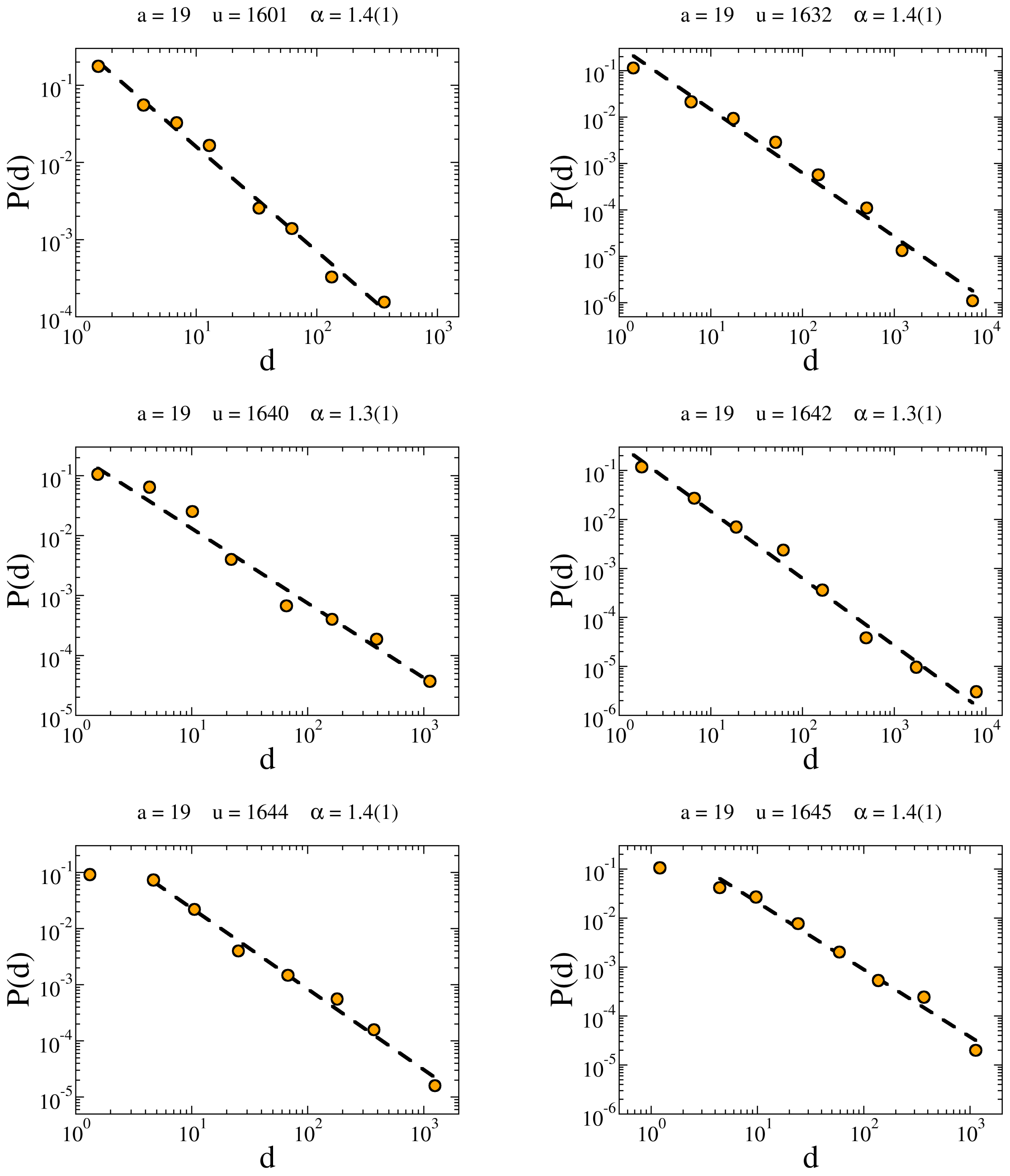

Figure S6: UBH data set. Same as Figure S5.

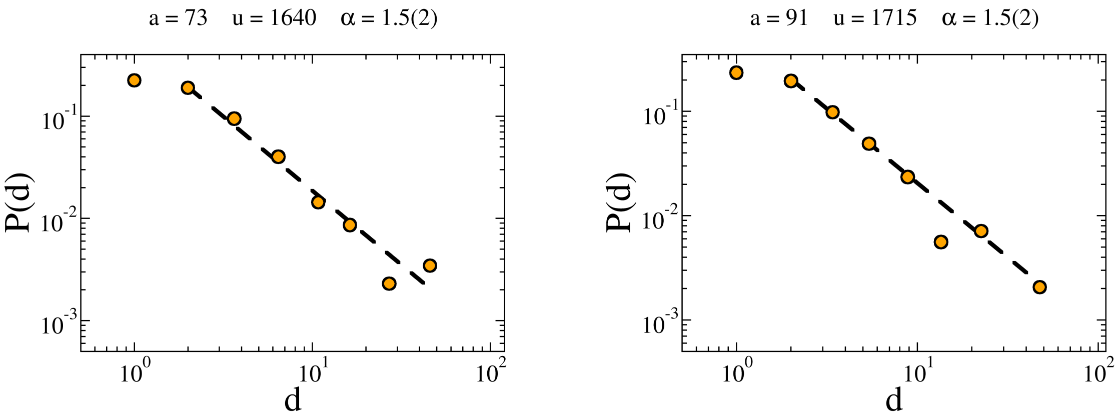

Figure S7: UBH data set. Same as Figure S5 and S6.

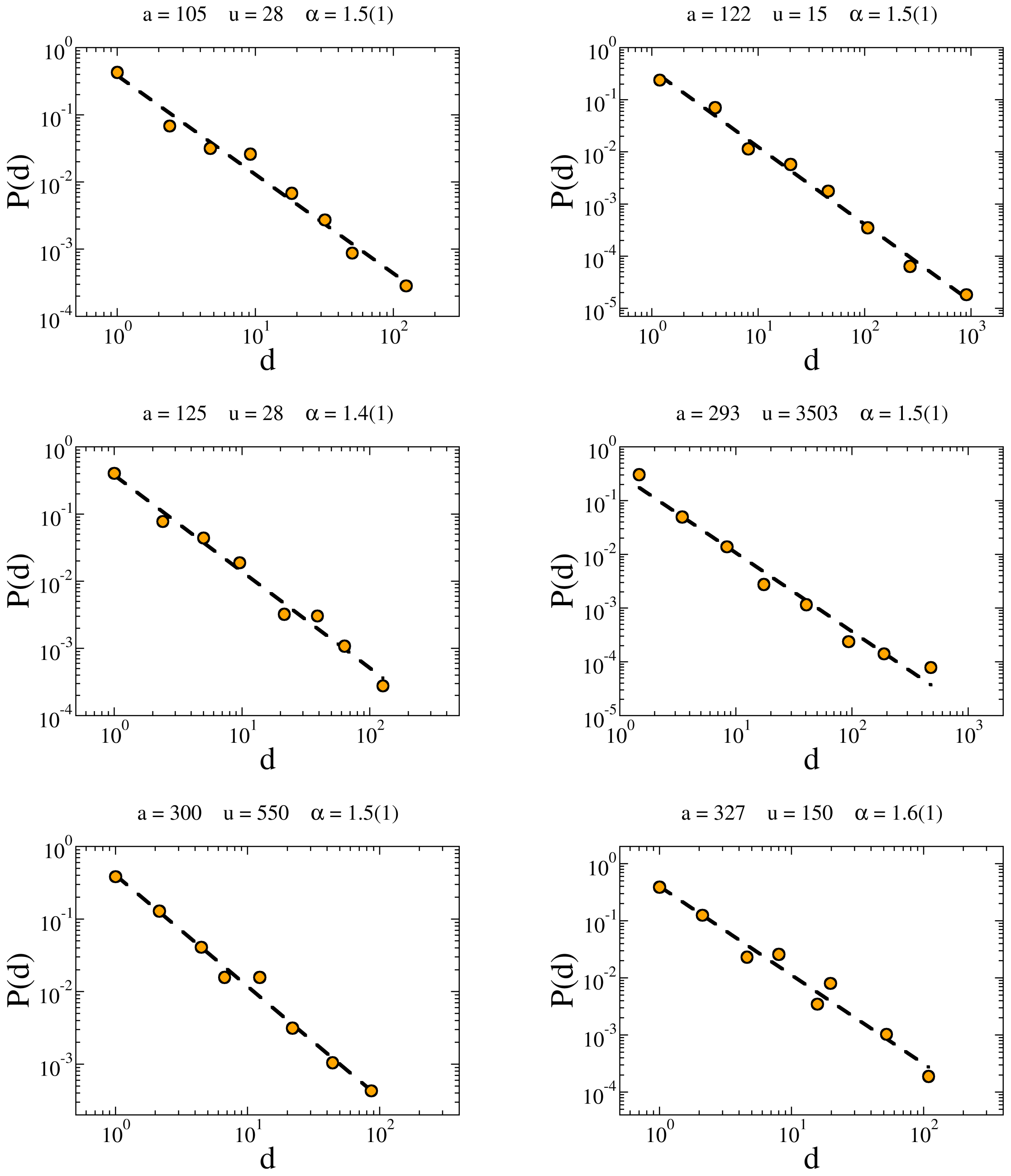

Figure S8: BM data set. Probability distribution function $P(d)$ measured for agent u in auction a. We show several $P(d)$s for different pairs u and a.

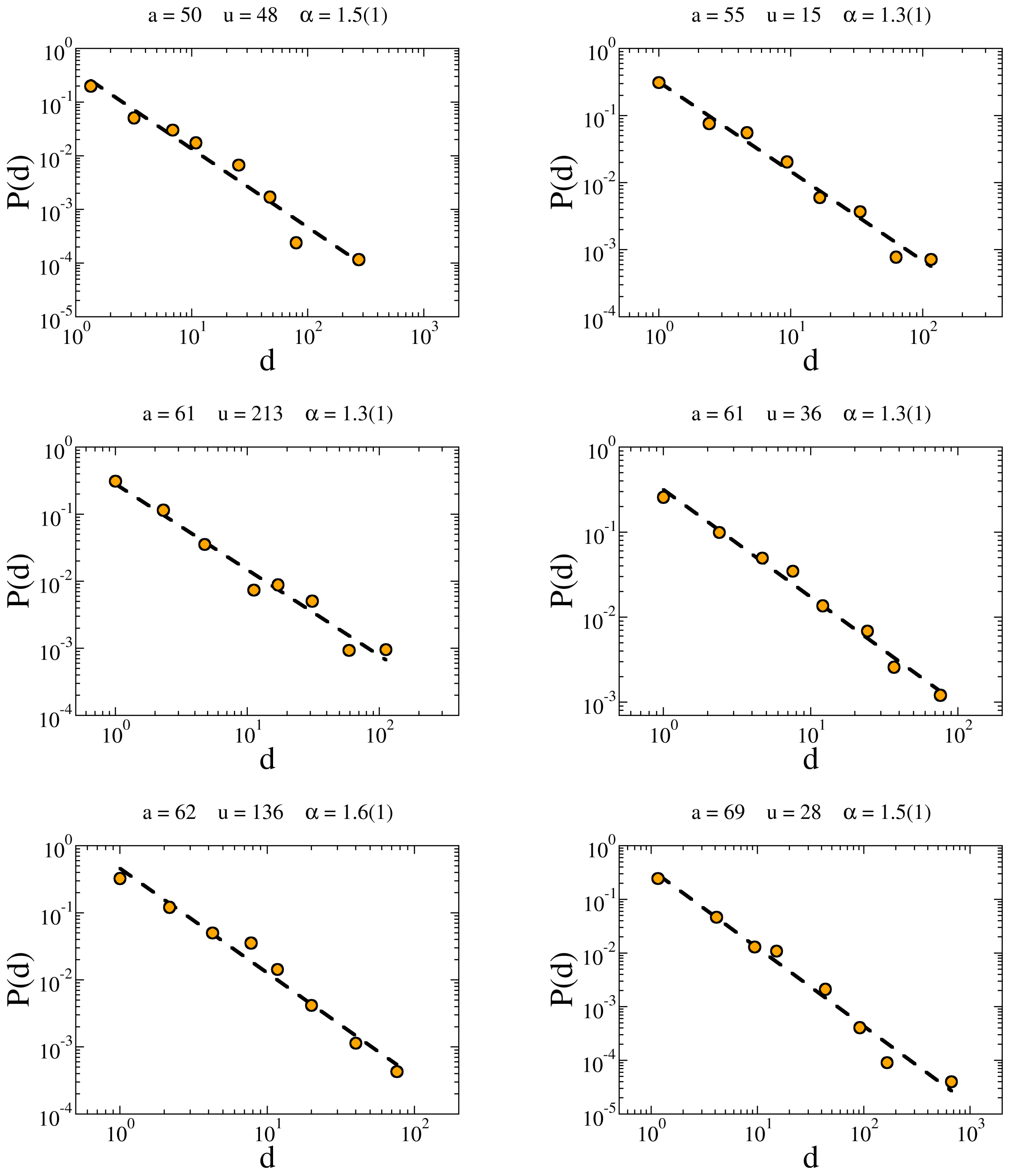

Figure S9: BM data set. Same as Figure S8.

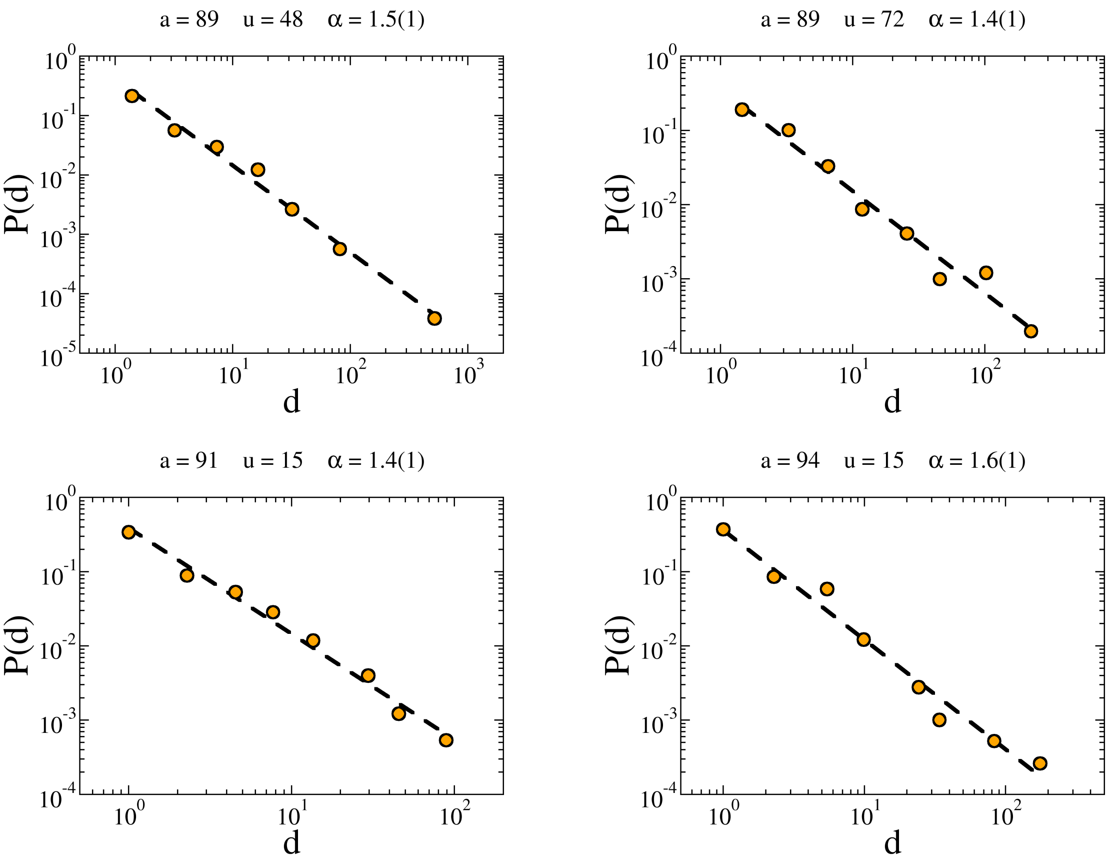

Figure S10: BM data set. Same as Figure S8 and S9.

S 1.6 Independence of the direction of the jumps

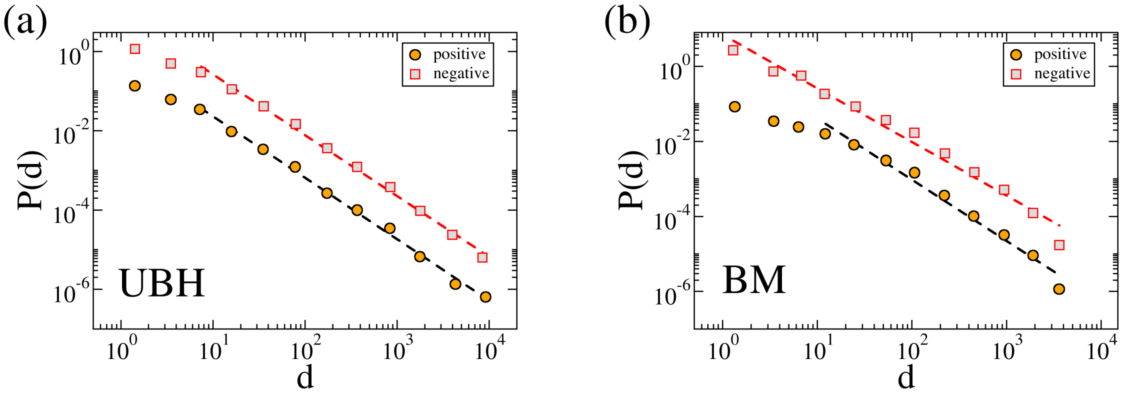

Figure S11: (a) UBH data set. Probability distribution function $P(d)$ calculated over all agents and auctions. We have separated positive from negative variations. The measured decay exponents of the best fits with power-laws (dashed lines) are $\alpha = 1.53(2)$ [black] and $\alpha = 1.54(2)$ [red], respectively. The curve corresponding to negative variation has been vertically shifted for clarity. This figure appears also in Fig. 3B of the main text. (b) BM data set. here the best power-law fit are $\alpha = 1.61(8)$ [black] and $\alpha = 1.45(4)$ [red].

Since the rules of the auction naturally bring agents to move towards low bid values, it is important to stress any eventual difference between the statistics associated with the length of gaps between consecutive bid values. We aggregated data from all agents and auctions and separate positive (i.e., $b_{t+1} > b_t$) from negative (i.e., $b_{t+1} < b_t$) variations. In Figure S11 the pdfs of positive and negative variations are plotted together. As one may notice, there is not a significant difference between them and both show a clear power-law decay with compatible exponents.

S 1.7 Independence of the agents' activity

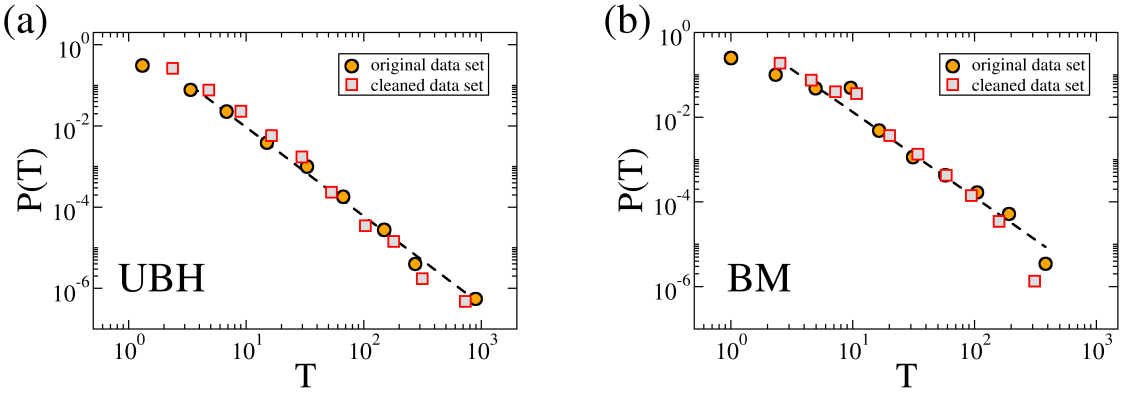

Figure S12: (a) UBH data set. Probability distribution function $P(T)$ of the number of bids T performed by single agents in single auctions. We calculate $P(T)$ on the original data set (orange circles) and on the cleaned version of the same data set (gray squares). In both cases, the distribution scales power-like with a decay exponent equal to $2.2(2)$. (b) BM data set. The exponent of the best fit is $2.0(4)$ [dashed line].

The evidence of Lévy flights for single agents in single auctions can be directly verified only for agents with a sufficient number of bids T in the same auction. For values of T smaller than 50 is practically impossible to construct the histogram $P(d)$ and therefore no exponent can be measured. Unfortunately, this situation is very frequent in our data sets. We measure the number of bids made each agent in each auction and plot the pdf of the number of bids in a single auction in Figure S12. The level of activity is quite heterogeneous and decays power-like with an exponent close to 2.2. For completeness, we measure the same pdf for both original and cleaned data sets without noticing appreciable differences.

We divide the population in different ranges of activity. We aggregate the length of the jumps performed by all agents in a given bin and measure the resulting $P(d)$. The results of this analysis are reported in Figure S13. Independently of the activity level, the aggregated pdfs

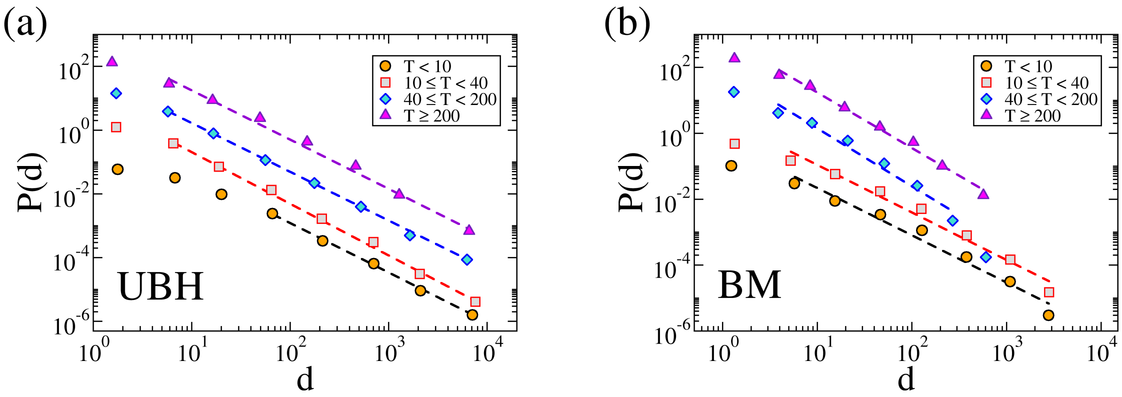

Figure S13: (a) UBH data set. Probability distribution function $P(d)$ of the length d of the jumps performed by agents in the bid space. All auctions have been aggregated together. Different curves correspond to agents with different levels of activity. Their activity is measured as the number of bids made in the same auction. We divide the population into four subsets: $T < 10$ (orange circles), $10 \leq T < 40$ (gray squares), $40 \leq T < 200$ (blue diamonds) and $T \geq 200$ (violet triangles). Dashed lines represent the best power-law fits. The value of the measured exponents are: $\alpha = 1.55(2)$ (black), $\alpha = 1.62(4)$ (red), $\alpha = 1.53(2)$ (blue) and $\alpha = 1.54(3)$ (violet). Curves have been vertically shifted for clarity. This figure is also reported in Fig. 3C of the main text. (b) BM data set. Best power-law fits (dashed lines) have exponents: $\alpha = 1.43(4)$ (black), $\alpha = 1.44(5)$ (red), $\alpha = 1.7(1)$ (blue) and $\alpha = 1.7(1)$ (violet).

decay power-like and have similar exponents α. This means that the presence of Lévy flights is typical for every agent independently of how many bids the agent has made.

S 1.8 Independence of the bidding time

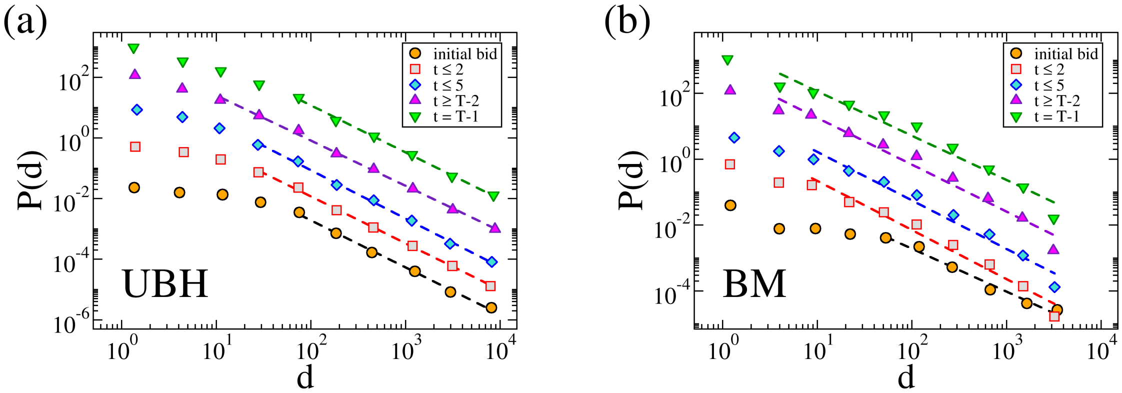

Figure S14: (a) UBH data set. Probability distribution function $P(d)$ of the length d of the flights performed by agents in the bid space. All auctions have been aggregated together. Different curves correspond to different periods of activity for agents: orange circles correspond to the initial guess made by agents; bids gaps with $t \leq 2$ (gray squares) and $t \leq 5$ (blue diamonds) aggregate the data corresponding to the early activity of agents; $t \geq T-2$ (violet up triangles) and $t = T-1$ (green down triangles) corresponds to the jumps made by agents at the end of their own activity. Dashed lines have been obtained as best power-law fits with data points. The value of the measured exponents are: $\alpha = 1.55(4)$ (black), $\alpha = 1.54(3)$ (red), $\alpha = 1.59(4)$ (blue), $\alpha = 1.49(5)$ (violet) and $\alpha = 1.54(6)$ (green). Curves have been vertically shifted for clarity. This figure is also reported in Fig. 2d of the main text. (b) BM data set. Best power-law fits (dashed lines) have exponents: $\alpha = 1.3(1)$ (black), $\alpha = 1.50(5)$ (red), $\alpha = 1.47(4)$ (blue), $\alpha = 1.43(6)$ (violet) and $\alpha = 1.35(6)$ (green). For the initial bid b_1, we compute the length of the jump as $d = M - b_1 + 1$, with M being the maximal bid value in the HUB auctions.

Another fundamental point is to understand whether the Lévy flight strategy is emergent or *a priori* given. We test these hypotheses by measuring the pdfs $P(d)$ corresponding to a certain range during the activity of the agents. The results are reported in Figure S14. We consider ranges of activity periods corresponding to $t \leq 2$, $t \leq 5$, $t \geq T-2$ and $t = T-1$. Additionally, we consider the distribution of the initial bid values (i.e., the first bid made by all agents in all auctions). In every case, we are able to fit the curves with power-laws and the resulting

exponents are compatible each other. We can effectively conclude that the strategy to adopt a Lévy flight is not an emergent property induced by the evolution of the auction. Instead the strategy to follow Lévy flights is intrinsically present, in each agent, during the whole duration of the auction.

S 1.9 Independence between jumps

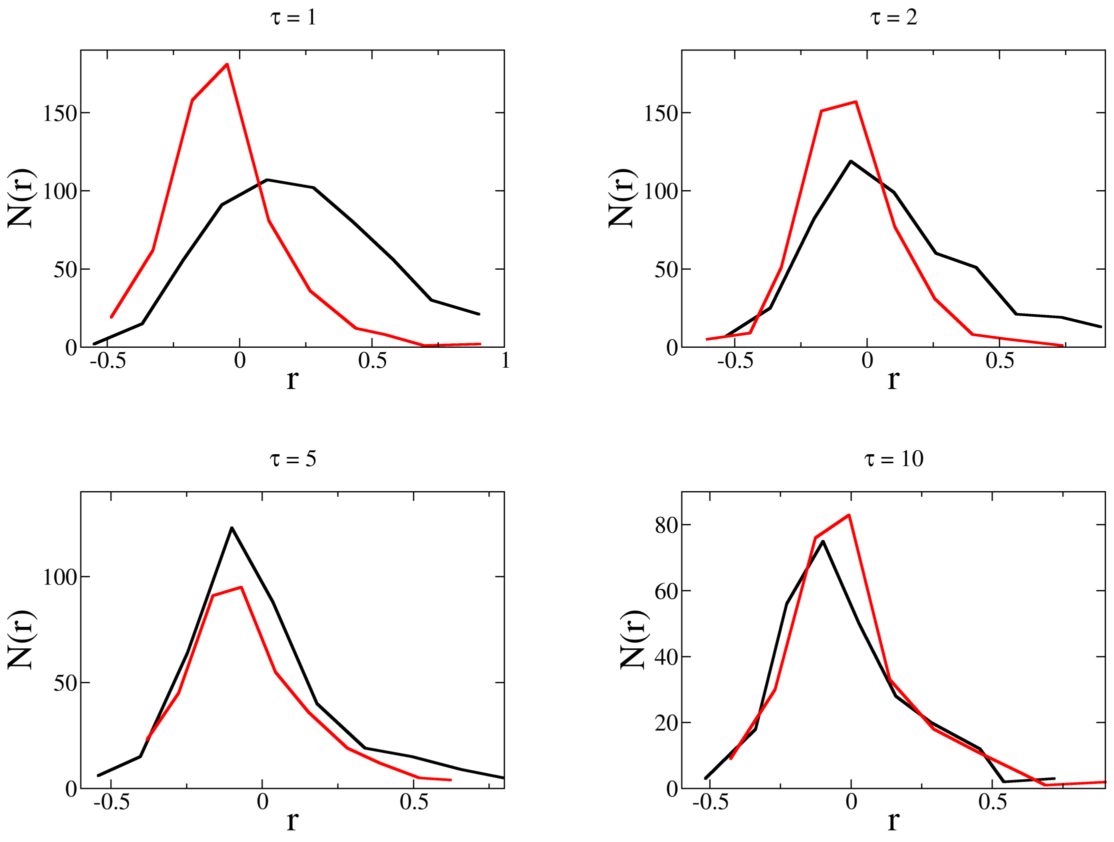

Figure S15: UBH data set. Number of agents $N(r)$ whose bid gaps at position t and $t+\tau$ have Pearson's correlation coefficient equal to r. Black curves are measured on real data, while the red ones are calculated over a reshuffled version of the same data. The reshuffling is made by randomly exchange pairs of entries in the time series of the time gaps with the only prescription that the sum of them is not lower than one and not larger than M. We consider different values of τ. In each plot only agents with at least $10+\tau$ bids in the same auction are considered.

We further study the correlations between jumps. Given an agent and an auction, we consider the list of all her jumps $d_1, d_2, \ldots, d_{B-1}$ and calculate the Pearson's correlation coefficient

$$r_\tau = \frac{\langle (d_t - \mu_t)\,(d_{t+\tau} - \mu_{t+\tau})\rangle}{\sigma_t\, \sigma_{t+\tau}} \quad , \tag{S2}$$

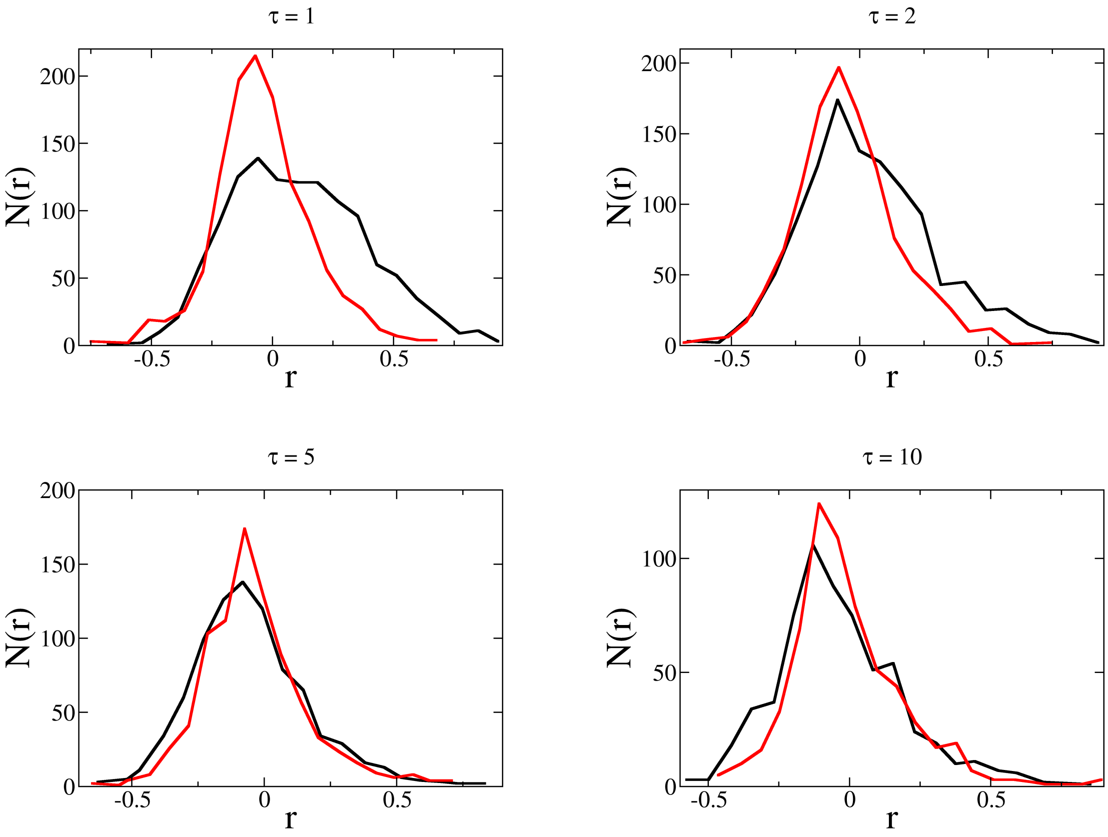

Figure S16: Same as those appearing in Figure S15 but for BM data set.

where $\langle \cdot \rangle$ stands for the average over the entire time series (i.e., over all values of t from 1 to $T-1-\tau$). $\mu_t = \langle d_t \rangle$ and $\mu_{t+\tau} = \langle d_{t+\tau} \rangle$ are the average values of the bid gaps along the time series, while $\sigma_t = \sqrt{\langle d_t^2 \rangle - \langle d_t \rangle}$ and $\sigma_{t+\tau} = \sqrt{\langle d_{t+\tau}^2 \rangle - \langle d_{t+\tau} \rangle}$ are the respective standard deviations. We measure such coefficient for every agent who has performed at least $10+\tau$ bids in the same auction and show the number of agents $N\left(r\right)$ with given value of r in Figures S15 and S16. The same quantity is also calculated for a randomized version of the time series, where bid gaps are randomly reshuffled with the only constraint that their partial sum cannot never be smaller than one and larger than M. We consider several values of τ. As one can

clearly notice, subsequent gaps (i.e., $\tau = 1$) are slightly correlated. Such correlation, becomes negligible when τ grows and already for $\tau = 2$, $N\left(r\right)$ is negligible. For $\tau = 5$ and $\tau = 10$, the curves corresponding to the original time series and those obtained over randomly reshuffled time series are almost identical.

Such results show that agents perform almost uncorrelated Lévy flights. Once an agent makes a jump, the length of this jump is slightly correlated with the one of the jump made before. However, after few jumps there is not longer memory of what happened before. In good approximation, the walk of the agent in the bid space can be therefore modeled as the one followed by a random walker performing uncorrelated Lévy flights.

S 1.10 Testing the model

S 1.10.1 Maximum likelihood fit and Goodness of fit

In this section, we compute the level of significance of our model for the description of real time series. Suppose that a time series of T bid values $b_1, b_2, \ldots, b_T$ describes a realization of our model. Fixed the exponent α, the bound M of the lattice and the position b_{t-1} at stage $t-1$, the probability that the random walker jumps at b_t at stage t is given by the transition matrix of Eq. (6) of the main text. The probability or *likelihood* that the whole sequence was extracted from our model is

$$p\left(b_1, b_2, \ldots, b_T \,|\alpha\right) = \left(Q_\alpha\right)_{0,b_1} \; \left(Q_\alpha\right)_{b_1,b_2} \; \left(Q_\alpha\right)_{b_2,b_3} \; \cdots \; \left(Q_\alpha\right)_{b_{T-1},b_T} \; .$$

The value of α that maximizes the former equation represents the most likely exponent of our model that could have generated our particular sequence. In order to find its maximum, it is convenient to take the logarithm of both sides and write the log-likelihood

$$\mathcal{L}\left(b_1, b_2, \ldots, b_T \,|\alpha\right) = \sum_{t=1}^{T} \ln\left[\left(Q_\alpha\right)_{b_{t-1},b_t}\right] = -\alpha \sum_{t=1}^{T} \ln\left|b_t - b_{t-1}\right| - \sum_{t=1}^{T} \ln\left[m_{b_{t-1}}\left(\alpha\right)\right] \; , \quad \text{(S3)}$$

where we set $b_0 = 0$. The value α' at which the maximum of Eq. (S3) occurs can be estimated numerically.

α' is the best exponent fitting the data in the hypothesis that they were produced according to our model. The significance level of the model for the description of the data can be calculated by estimating the p-value associated with our measurement. In this respect, we first compute the distance between the theoretical distribution of the jump lengths

$$P_{\alpha'}\left(d\right) = \left[\sum_{i,j} \left(Q_{\alpha'}\right)_{ij} \delta\left(d - |i-j|\right)\right] \; \left[\sum_{d}\sum_{i,j} \left(Q_{\alpha'}\right)_{ij} \delta\left(d - |i-j|\right)\right]^{-1}$$

and the one obtained from our data $P\left(d\right)$ by calculating

$$\eta_{data} = \max_{t} \left|P_{\alpha'}\left(\geq d_t\right) - P\left(\geq d_t\right)\right| \quad ,$$

where $P(\geq d) = \sum_{q \geq d} P(q)$ is the cumulative distribution of the jump lengths. Notice that the distance between cumulative distributions is the same as the one adopted in the Kolmogorov-Smirnov test. We then generate artificial time series of length T from our model with exponent α' and compute their distance η with respect to the theoretical distribution. The p-value is finally determined by the relative number of times in which we observe $\eta \geq \eta_{data}$.

Synthetic time series generated according to our model can be additionally used for the determination of the error associated to the estimation of α'. The error associated to α' is the standard deviation of the best exponents estimated, with maximum likelihood, for the synthetic data sets.

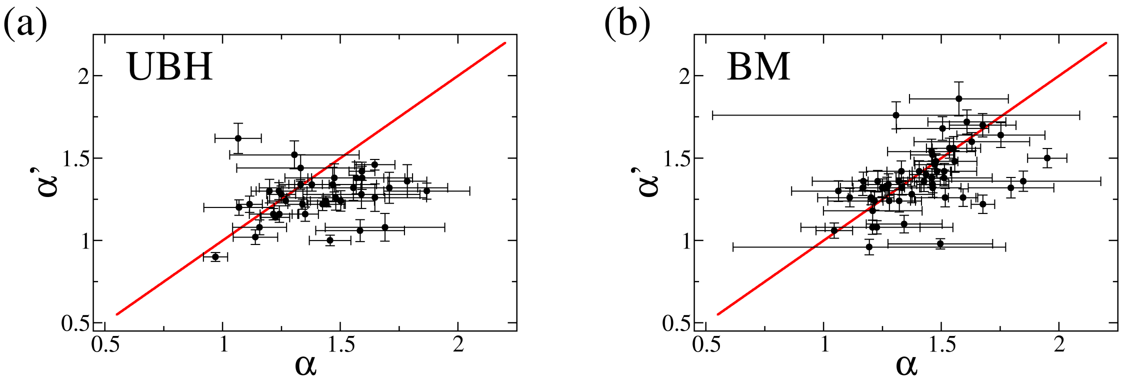

Figure S17: We consider only agents who have performed at least $T = 50$ bids in a single auction in UBH data set and those with at least $T = 100$ in the BM data set. Our data set offers 39 agents that satisfy this constraint in the UBH data set and 52 in the BM data set. Panels a (UBH) and b (BM) show the scatter plot α' *versus* α for the best power-law exponents estimated by using maximum likelihood and least square methods, respectively. The agreement between the two measurements is good as demonstrated by the fact that the majority of the points fall on the diagonal (red line).

A graphical comparison between the exponents α (least square method) and α' (maximum likelihood method) is presented in Figure S17. In general, the two methods produce consistent results. For completeness, we list the results obtained in Tables S2 and S3.

a	u	α	α'	p
1	23	1.6(1)	1.5(1)	0.00
1	81	1.2(1)	1.3(1)	0.17
100	1715	1.6(2)	1.1(1)	0.01
100	81	1.5(2)	1.3(1)	0.00
104	3093	1.7(3)	1.1(1)	0.14
108	134	1.7(1)	1.3(1)	0.02
14	134	1.2(1)	1.1(1)	0.03
14	81	1.9(2)	1.3(1)	0.00
15	423	1.6(4)	1.3(1)	0.3
179	3663	1.8(1)	1.4(1)	0.17
19	1	1.3(1)	1.3(1)	0.28
19	1313	1.2(1)	1.2(1)	0.14
19	134	1.2(1)	1.1(1)	0.00
19	1433	1.1(1)	1.0(1)	0.06
19	1448	1.4(1)	1.2(1)	0.01
19	1558	1.1(1)	1.2(1)	0.63
19	1576	1.0(1)	0.9(1)	0.35
19	1601	1.4(1)	1.3(1)	0.01
19	1632	1.4(1)	1.2(1)	0.01
19	1640	1.3(1)	1.2(1)	0.00

a	u	α	α'	p
19	1642	1.3(1)	1.2(1)	0.13
19	1644	1.4(1)	1.2(1)	0.00
19	1645	1.4(1)	1.2(1)	0.00
19	3	1.2(1)	1.3(1)	0.9
19	363	1.2(1)	1.1(1)	0.02
19	434	1.1(1)	1.2(1)	0.35
19	438	1.5(1)	1.0(1)	0.02
20	617	1.6(2)	1.4(1)	0.01
22	134	1.2(1)	1.3(1)	0.95
44	433	1.1(1)	1.6(1)	0.07
46	2003	1.3(3)	1.5(1)	0.02
5	128	1.6(1)	1.4(1)	0.00
62	2392	1.3(3)	1.4(1)	0.43
71	324	1.6(2)	1.4(1)	0.00
73	1640	1.5(2)	1.4(1)	0.18
73	1715	1.5(1)	1.2(1)	0.34
79	134	1.6(2)	1.3(1)	0.11
91	1715	1.5(2)	1.3(1)	0.32
97	1715	1.6(2)	1.3(1)	0.09

Table S2: UBH data set. Each row corresponds to one of the 39 agents who have bid at least 50 times in the same auction. We report the id of the auction a, the id of the agent u, the exponent α calculated with the least square method, the exponent α' calculated with the maximum likelihood method and the p-value. Entries with low p-values are highlighted in gray. In the 77% of the cases we find a p-value larger than 0, which indicates that our model well describe the time series.

The p-values show also a general goodness of our model for the description of the data. Sometimes however, the value of p is very small. This could be explained in a simple manner. In Figure S18 we plot for example the statistical test performed over the same time series appearing in Figure S2. In that auction, the maximum bid amount was $M = 13\,000$. However, this value of M does not correspond to the "effective" bound felt by the agent. This bound seems to be around $M \sim 2\,000$ as the sudden drop of $P\,(\geq d)$ would suggest. By setting $M = 2\,200$ and running again the statistical test, as we did in Figure S18b, we see clearly that the curve predicted by our model and the one measured on real data are in very good agreement.

a	u	α	α'	p
11	28	1.1(2)	1.3(1)	0.09
13	48	1.5(1)	1.3(1)	0
13	28	1.3(1)	1.4(1)	0.04
15	11	1.8(3)	1.4(1)	0.05
15	36	2.0(1)	1.5(1)	0.03
28	28	1.4(1)	1.4(1)	0.58
32	150	1.2(6)	1.0(1)	0
39	48	1.3(1)	1.3(1)	0
47	28	1.1(1)	1.3(1)	0.48
50	48	1.5(1)	1.3(1)	0.27
52	28	1.2(2)	1.4(1)	0.01
55	15	1.3(1)	1.3(1)	0.38
55	150	1.2(3)	1.1(1)	0
61	213	1.3(1)	1.2(1)	0.62
61	36	1.3(1)	1.2(1)	0.5
62	136	1.6(1)	1.5(1)	0.2
67	98	1.0(1)	1.1(1)	0.15
69	28	1.5(1)	1.4(1)	0.39
82	36	1.2(2)	1.2(1)	0
89	72	1.4(1)	1.3(1)	0.15
89	48	1.5(1)	1.3(1)	0.2
89	28	1.2(1)	1.3(1)	0.16
91	15	1.4(1)	1.4(1)	0.65
92	36	1.8(2)	1.3(1)	0
92	28	1.3(1)	1.3(1)	0.42
94	48	1.6(1)	1.3(1)	0
94	98	1.5(2)	1.0(1)	0
94	15	1.5(1)	1.5(1)	0.15
105	28	1.5(1)	1.5(1)	0.45
110	28	1.2(1)	1.4(1)	0.18
116	15	1.2(1)	1.3(1)	0.04
122	15	1.5(1)	1.4(1)	0.78
125	28	1.4(1)	1.4(1)	0.88
133	98	1.7(1)	1.2(1)	0.02
162	15	1.2(1)	1.3(1)	0.48
181	15	1.2(2)	1.1(1)	0.1
197	15	1.2(2)	1.2(1)	0.58
201	15	1.3(2)	1.1(1)	0
262	150	1.3(8)	1.8(1)	0.04
264	1428	1.5(2)	1.7(1)	0.12
269	122	1.6(2)	1.7(1)	0.02
279	550	1.5(2)	1.5(1)	0.22
293	3503	1.5(1)	1.5(1)	0
300	550	1.5(1)	1.6(1)	0.41
300	150	1.7(1)	1.7(1)	0.17
306	150	1.5(1)	1.4(1)	0
317	150	1.8(2)	1.6(1)	0.22
318	150	1.5(2)	1.4(1)	0.04
327	1503	1.3(1)	1.4(1)	0.19
327	150	1.6(1)	1.6(1)	0.25
331	150	1.6(2)	1.6(1)	0.29
332	1574	1.6(2)	1.9(1)	0.15

Table S3: BM data set. Each row corresponds to one of the 52 agents who have bid at least 100 times in the same auction. We report the id of the auction a, the id of the agent u, the exponent α calculated with the least square method, the exponent α' calculated with the maximum likelihood method and the p-value. Entries with low p-values are highlighted in gray. In the 79% of the cases we find a p-value larger than 0, which indicates that our model well describe the time series.

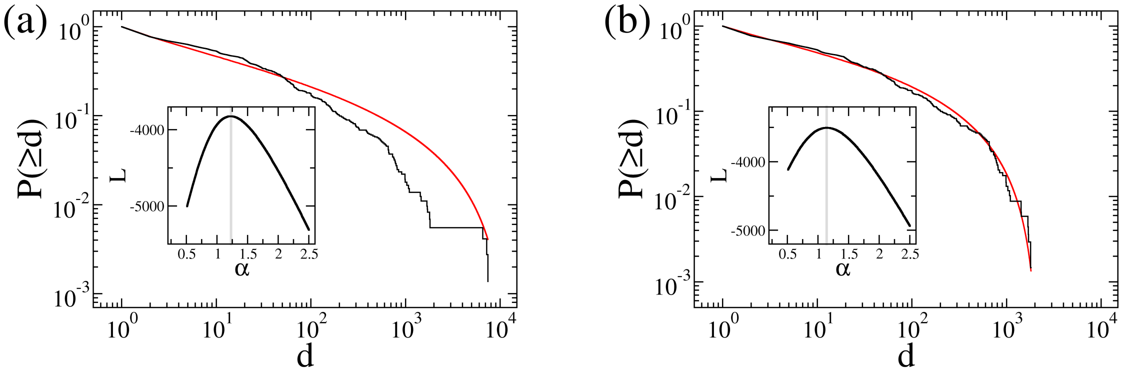

Figure S18: Example of the maximum likelihood method for the determination of the exponent α' of the Lévy flight. We consider the time series of agent 1632 in auction 19 of the UBH data set (the same appearing in Figure S2). In panel a, we set $M = 13\,000$ which is the value of the maximum bid amount that was allowed in the auction. The best exponent $\alpha' = 1.2(1)$ is obtained by looking at the maximum of the log-likelihood as it is shown in the inset. The comparison between the cumulative distribution of the jump lengths expected from the model (red line) and the one calculated over the time series (black) do not well agree. It seems that the "effective" bound is smaller than the real one. In panel b, we set $M = 2\,200$ and consider only bid values smaller than this bound. On the new time series, we perform a maximum likelihood fit finding $\alpha' = 1.1(1)$. The theoretical expectation (red line) and the one obtained from the time series (black line) are now very similar yielding a p-value equal to 0.1.

S 1.10.2 Another maximum likelihood fit

Since the upper-bound M is agent dependent, we perform an additional analysis where the upper bound M is not directly taken from the data, but used as a parameter for the fit. For each agent, we let the parameter M vary only in the range for which at least the 90% of the bids values are below M. Indicate with $\tilde{T}$ the number of bids below the threshold M. We then find α' identifying the maximum of the likelihood function and calculate the p-value as described so far. We consider the best value of M as the one which maximizes the product $\tilde{T} \times p$. Figures S19, S20 and S21 report the best fit for the same agents analyzed in Figures S5, S6 and S7 for UBH data set, while Figures S22, S23 and S24 are the analogous of Figures S8, S9 and S10 for BM data set. For completeness, we report in Tables S4 and S5 the results obtained with the maximum likelihood fit where M is used as parameter of the fit. The best exponents α' are still consistent with those reported in Tables S2 and S3, but the p-values result much increased.

a	u	α	α'	M	p
1	23	1.6(1)	1.4(1)	490	0.00
1	81	1.2(1)	1.2(1)	2220	0.26
100	1715	1.6(2)	1.0(1)	150	0.15
100	81	1.5(2)	1.3(1)	480	0.01
104	3093	1.7(3)	1.0(1)	70	0.51
108	134	1.7(1)	1.3(1)	70	0.09
14	134	1.2(1)	1.1(1)	530	0.40
14	81	1.9(2)	1.3(1)	870	0.01
15	423	1.6(4)	1.2(1)	120	0.53
179	3663	1.8(1)	1.3(2)	70	0.35
19	1	1.3(1)	1.3(1)	3050	0.29
19	1313	1.2(1)	1.1(1)	1650	0.50
19	134	1.2(1)	1.0(1)	2320	0.19
19	1433	1.1(1)	0.9(1)	1840	0.99
19	1448	1.4(1)	1.1(1)	2480	0.08
19	1558	1.1(1)	1.2(1)	2290	0.93
19	1576	1.0(1)	0.9(1)	9580	0.34
19	1601	1.4(1)	1.2(1)	480	0.09
19	1632	1.4(1)	1.1(1)	2210	0.13
19	1640	1.3(1)	1.2(1)	3080	0.00

a	u	α	α'	M	p
19	1642	1.3(1)	1.2(1)	3200	0.45
19	1644	1.4(1)	1.2(1)	3010	0.00
19	1645	1.4(1)	1.1(1)	1750	0.03
19	3	1.2(1)	1.3(1)	3310	0.91
19	363	1.2(1)	0.9(1)	890	0.20
19	434	1.1(1)	1.1(1)	1750	0.77
19	438	1.5(1)	0.9(1)	2120	0.43
20	617	1.6(2)	1.3(1)	200	0.09
22	134	1.2(1)	1.3(1)	1000	0.96
44	433	1.1(1)	1.6(1)	3340	0.08
46	2003	1.3(3)	1.4(1)	110	0.03
5	128	1.6(1)	1.4(1)	1830	0.01
62	2392	1.3(3)	1.3(1)	130	0.52
71	324	1.6(2)	1.4(1)	860	0.00
73	1640	1.5(2)	1.4(1)	1920	0.27
73	1715	1.5(1)	1.2(1)	140	0.68
79	134	1.6(2)	1.3(1)	120	0.21
91	1715	1.5(2)	1.3(1)	70	0.67
97	1715	1.6(2)	1.2(1)	80	0.19

Table S4: UBH data set. Each row corresponds to one of the 39 agents who have bid at least 50 times in the same auction. We report the id of the auction a, the id of the agent u, the exponent α calculated with the least square method, the exponent α' calculated with the maximum likelihood method, the best value of the upper bound M and the p-value. Entries with low p-values are highlighted in gray. In the 90% of the cases we find a p-value larger than 0, which indicates that our model well describe the time series.

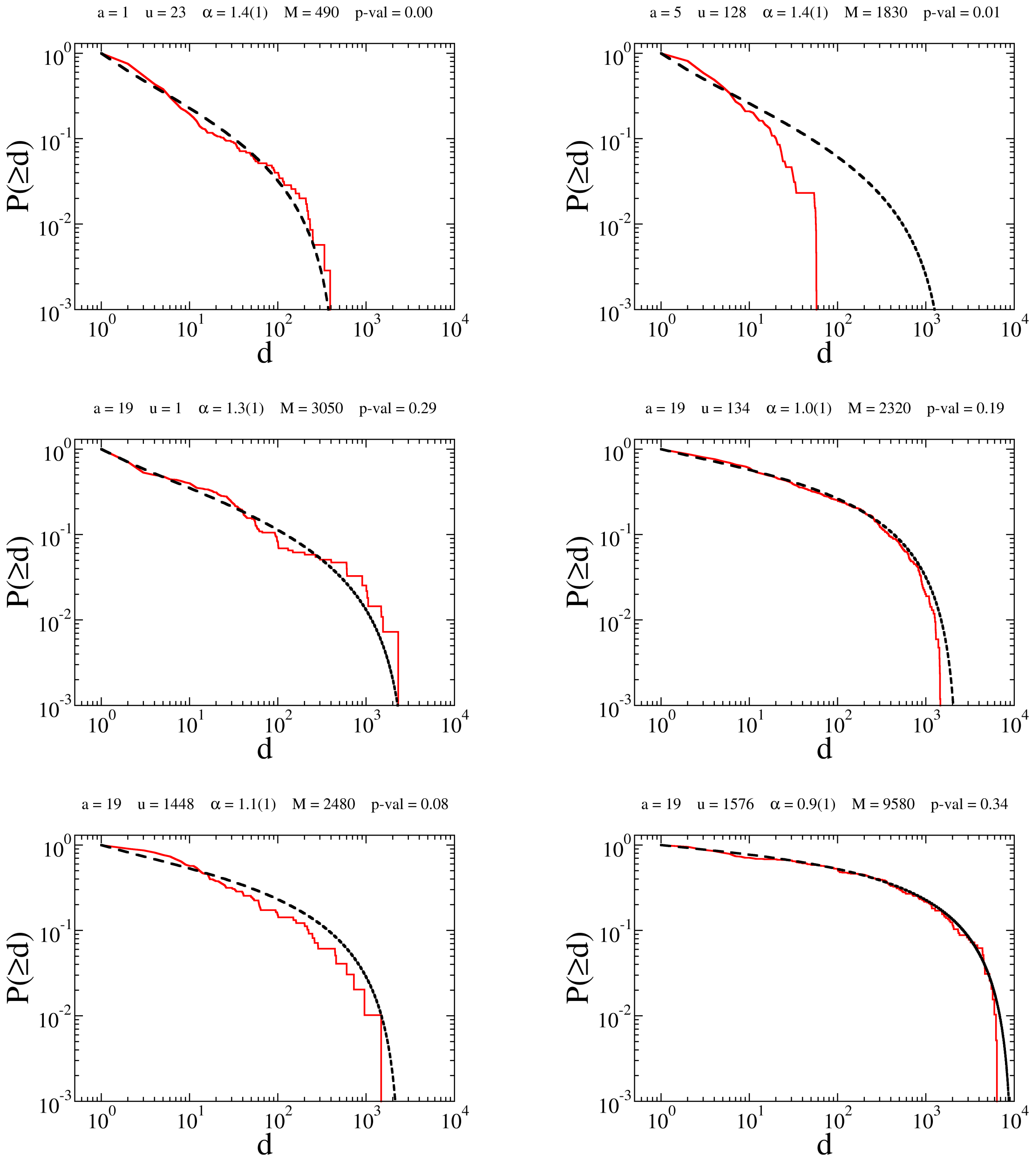

Figure S19: UBH data set. Cumulative distribution function $P\,(\geq d)$ measured for agent u in auction a (red full line) compared with the theoretical distribution (black dashed line). We show several $P\,(\geq d)$s for different pairs u and a. We report also the best value of the upper bound M and the p-value associated with our fit.

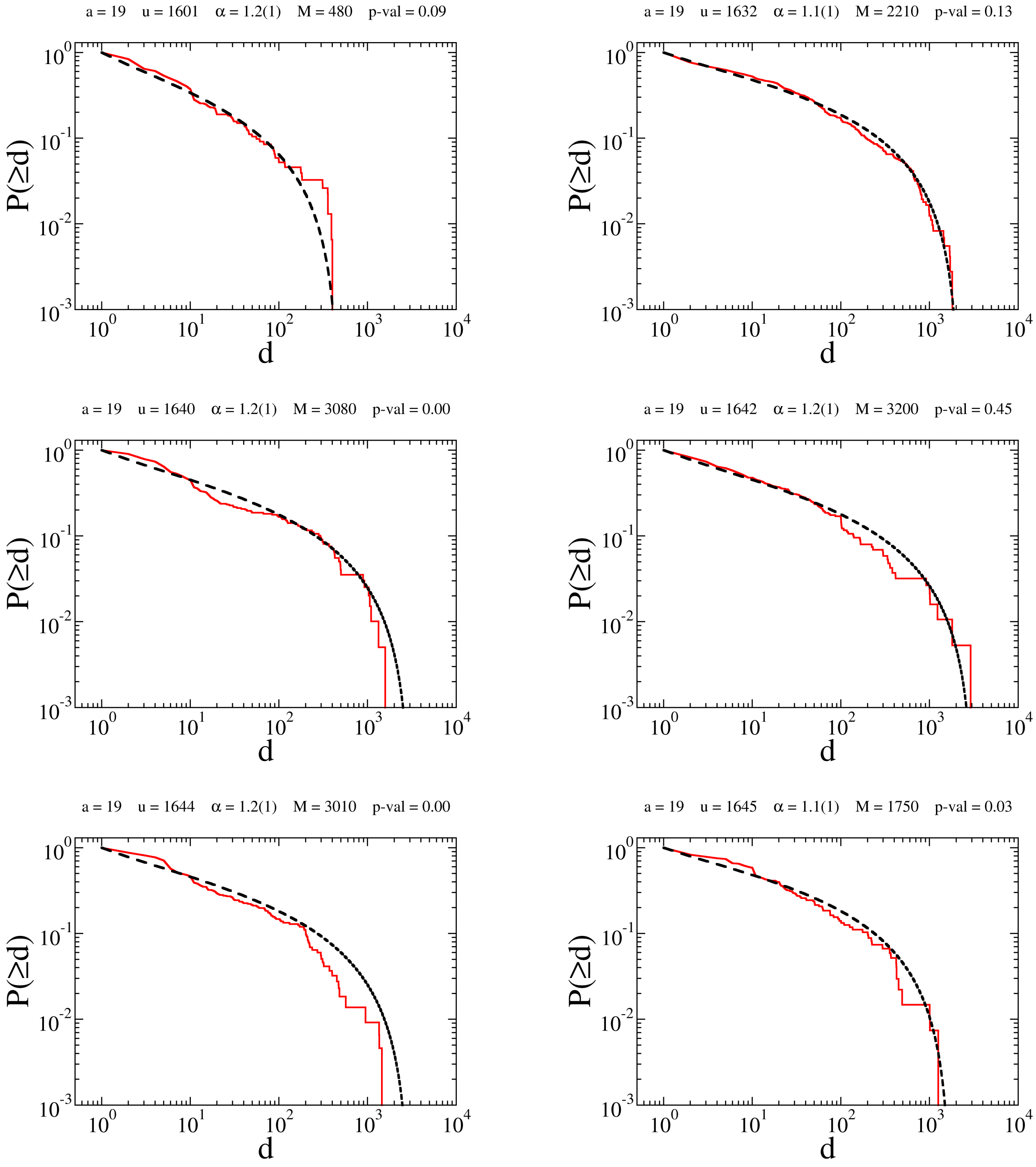

Figure S20: UBH data set. Same as Figure S19.

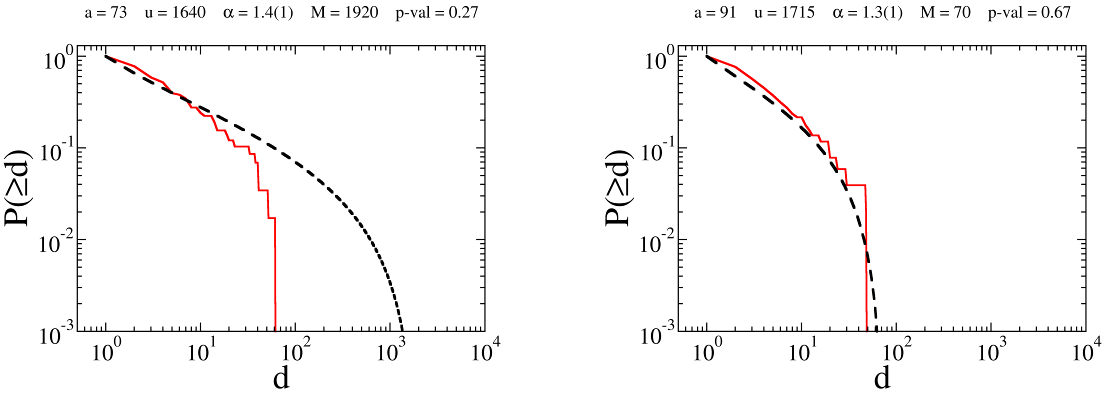

Figure S21: UBH data set. Same as Figure S19 and S20.

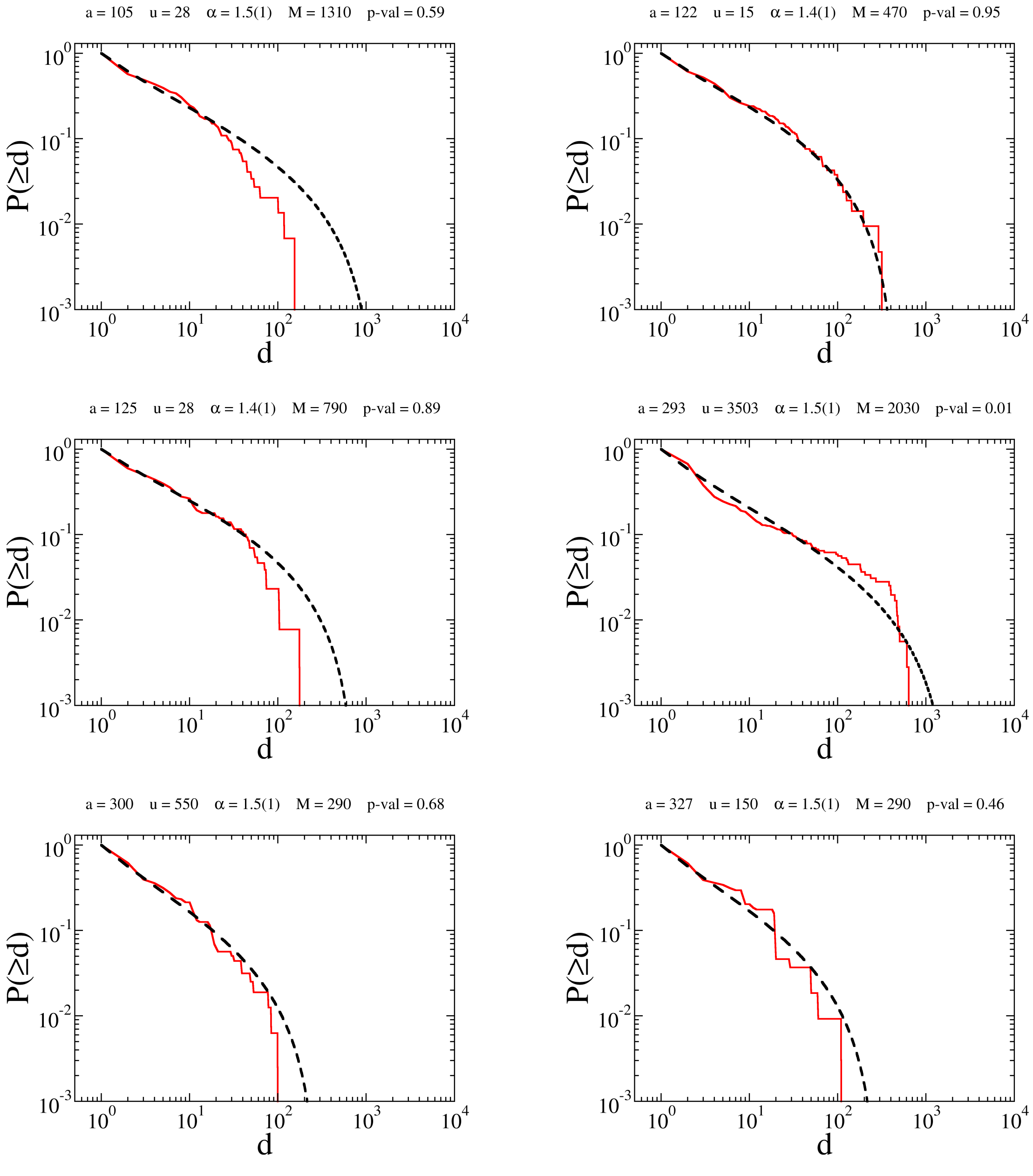

Figure S22: BM data set. Cumulative distribution function $P(\geq d)$ measured for agent u in auction a (red full line) compared with the theoretical distribution (black dashed line). We show several $P(\geq d)$s for different pairs u and a. We report also the best value of the upper bound M and the p-value associated with our fit.

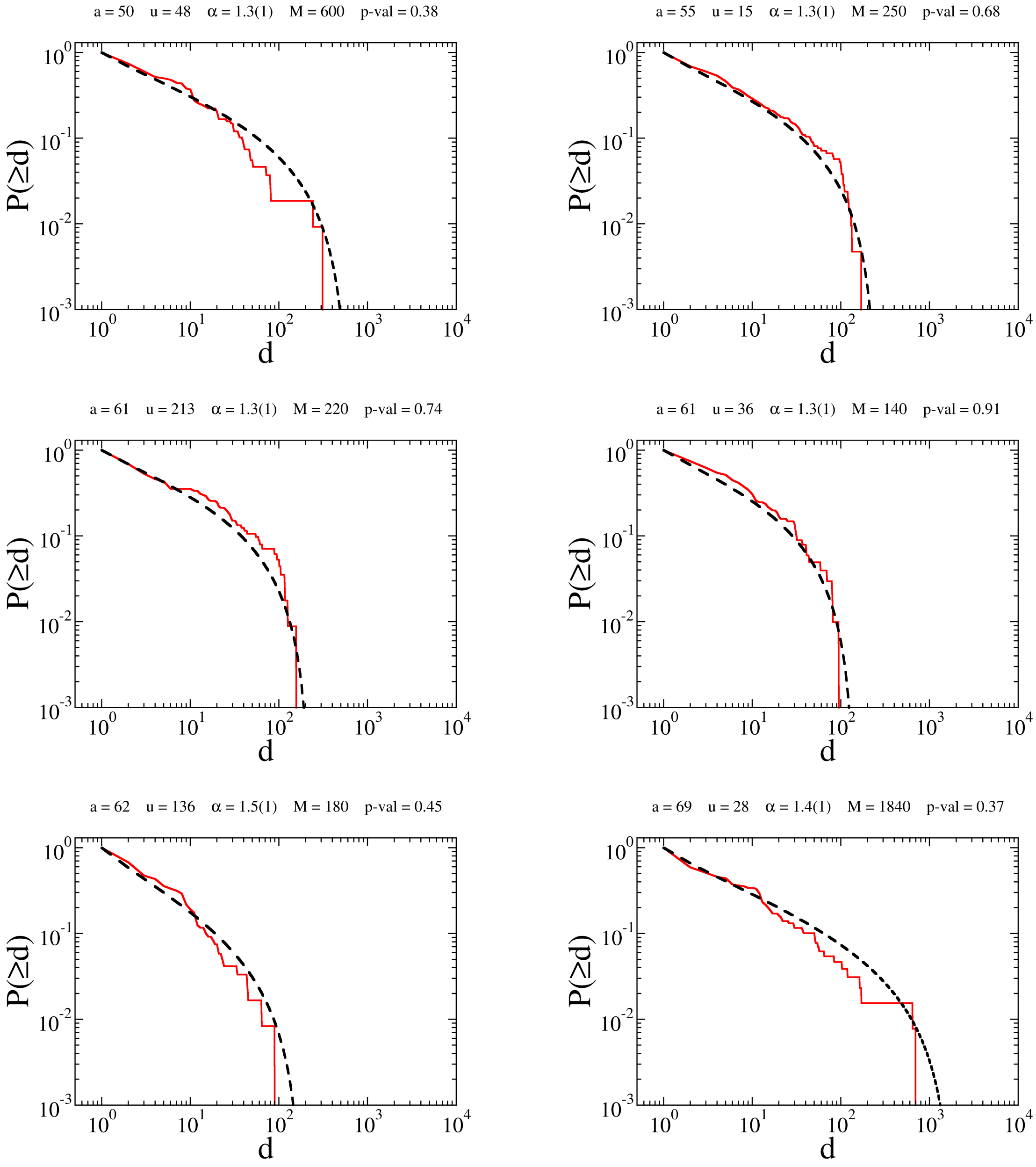

Figure S23: BM data set. Same as Figure S22.

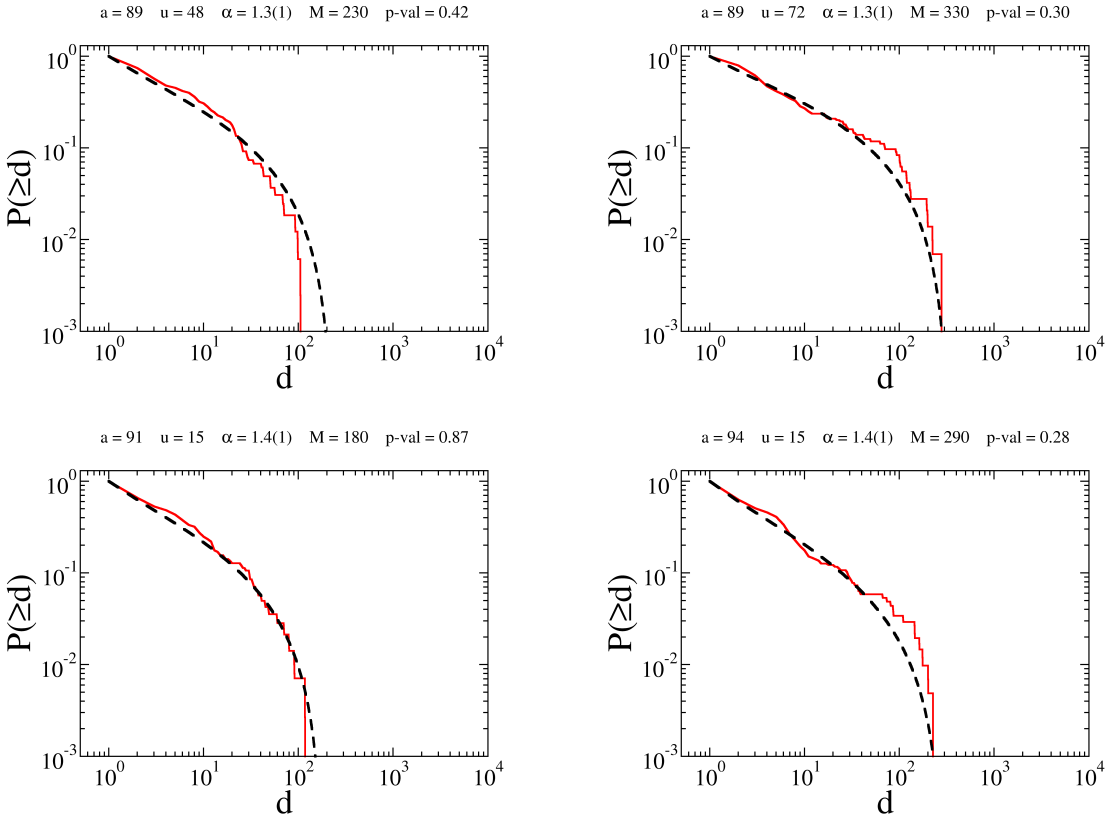

Figure S24: BM data set. Same as Figure S22 and S23.

a	u	α	α'	M	p
11	28	1.1(2)	1.4(1)	1290	0.23
13	48	1.5(1)	1.2(1)	570	0.00
13	28	1.3(1)	1.3(1)	310	0.15
15	11	1.8(3)	1.3(1)	180	0.23
15	36	2.0(1)	1.5(1)	200	0.19
28	28	1.4(1)	1.4(1)	530	0.53
32	150	1.2(6)	1.0(1)	160	0.00
39	48	1.3(1)	1.3(1)	170	0.02
47	28	1.1(1)	1.2(1)	320	0.59
50	48	1.5(1)	1.3(1)	600	0.38
52	28	1.2(2)	1.4(1)	2790	0.11
55	15	1.3(1)	1.3(1)	250	0.68
55	150	1.2(3)	1.1(1)	220	0.00
61	213	1.3(1)	1.3(1)	220	0.74
61	36	1.3(1)	1.2(1)	140	0.91
62	136	1.6(1)	1.5(1)	180	0.45
67	98	1.0(1)	1.0(1)	210	0.83
69	28	1.5(1)	1.4(1)	1840	0.37
82	36	1.2(2)	1.2(1)	360	0.01
89	72	1.4(1)	1.3(1)	330	0.30
89	48	1.5(1)	1.3(1)	230	0.42
89	28	1.2(1)	1.3(1)	1580	0.17
91	15	1.4(1)	1.4(1)	180	0.87
92	36	1.8(2)	1.3(1)	130	0.04
92	28	1.3(1)	1.4(1)	910	0.46
94	48	1.6(1)	1.2(1)	310	0.18

a	u	α	α'	M	p
94	98	1.5(2)	0.9(1)	280	0.03
94	15	1.5(1)	1.4(1)	290	0.28
105	28	1.5(1)	1.5(1)	1310	0.59
110	28	1.2(1)	1.4(1)	2870	0.36
116	15	1.2(1)	1.3(1)	3050	0.13
122	15	1.5(1)	1.4(1)	470	0.95
125	28	1.4(1)	1.4(1)	790	0.89
133	98	1.7(1)	1.2(1)	140	0.15
162	15	1.2(1)	1.4(1)	3270	0.54
181	15	1.2(2)	1.0(1)	250	0.34
197	15	1.2(2)	1.2(1)	180	0.79
201	15	1.3(2)	1.1(1)	110	0.43
262	150	1.3(8)	1.8(1)	1200	0.08
264	1428	1.5(2)	1.7(1)	1610	0.16
269	122	1.6(2)	1.7(1)	970	0.02
279	550	1.5(2)	1.6(1)	2600	0.32
293	3503	1.5(1)	1.5(1)	2030	0.01
300	550	1.5(1)	1.5(1)	290	0.68
300	150	1.7(1)	1.7(1)	1900	0.26
306	150	1.5(1)	1.4(1)	740	0.00
317	150	1.8(2)	1.6(1)	260	0.38
318	150	1.5(2)	1.4(1)	610	0.05
327	1503	1.3(1)	1.4(1)	230	0.54
327	150	1.6(1)	1.5(1)	290	0.46
331	150	1.6(2)	1.6(1)	1500	0.42
332	1574	1.6(2)	1.9(1)	1140	0.15

Table S5: BM data set. Each row corresponds to one of the 52 agents who have bid at least 100 times in the same auction. We report the id of the auction a, the id of the agent u, the exponent α calculated with the least square method, the exponent α' calculated with the maximum likelihood method, the best value of the upper bound M and the p-value. Entries with low p-values are highlighted in gray. In the 92% of the cases we find a p-value larger than 0, which indicates that our model well describe the time series.

S 1.10.3 Probability distribution of the Lévy flight exponents

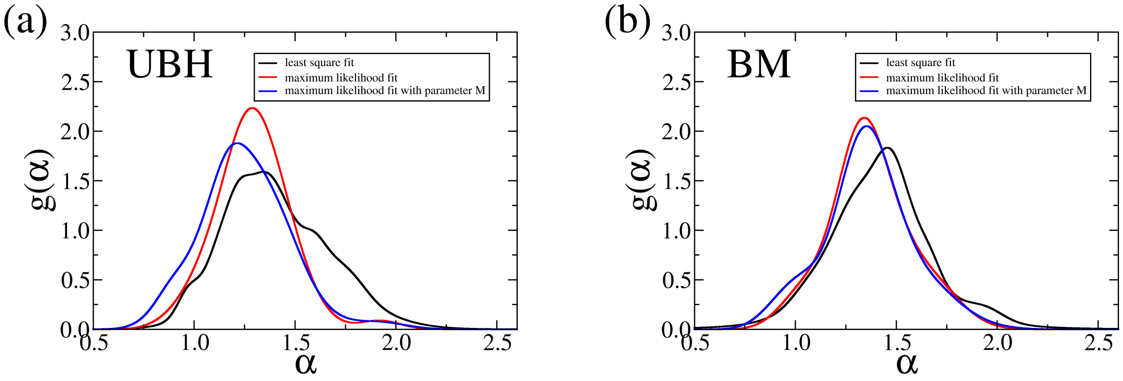

Figure S25: The pdf $g\left(\alpha\right)$ of the exponents calculated with the three fitting methods. The pdfs corresponding to the maximum likelihood fit, where M is a parameter of the fit, are the same as those appearing in Figs. 2D and 2E of the main text. The distributions are characterized by the following values of the mode α_b, average $\langle\alpha\rangle$ and variance σ. UBH data set: for least square fit we have $\alpha_b = 1.34$, $\langle\alpha\rangle = 1.40$, $\sigma = 0.26$; for maximum likelihood fit we have $\alpha_b = 1.28$, $\langle\alpha\rangle = 1.29$, $\sigma = 0.20$; for maximum likelihood fit with additional fitting parameter M we have $\alpha_b = 1.21$, $\langle\alpha\rangle = 1.26$, $\sigma = 0.23$. BM data set: for least square fit we have $\alpha_b = 1.46$, $\langle\alpha\rangle = 1.42$, $\sigma = 0.27$; for maximum likelihood fit we have $\alpha_b = 1.34$, $\langle\alpha\rangle = 1.37$, $\sigma = 0.21$; for maximum likelihood fit with additional fitting parameter M we have $\alpha_b = 1.35$, $\langle\alpha\rangle = 1.36$, $\sigma = 0.23$.

As a final result, we compute the distribution $g\left(\alpha\right)$ of the exponents measured for single agents in single auctions. We still consider only agents who have performed at least $T = 50$ bids in a single auction in UBH data set and those with at least $T = 100$ in the BM data set. Assuming that the best estimation of the exponent of agent u is α_u and the associated error of the measurement is $\Delta\alpha_u$, the empirical distribution of the exponents is calculated as

$$g\left(\alpha\right) = C^{-1} \sum_u \frac{1}{\Delta\alpha_u} \, e^{-(\alpha_u-\alpha)^2 / \left[2\,(\Delta\alpha_u)^2\right]} \quad , \tag{S4}$$

where $C = \int d\alpha \, \sum_u \frac{1}{\Delta\alpha_u} \, e^{-(\alpha_u-\alpha)^2 / \left[2\,(\Delta\alpha_u)^2\right]}$ is the proper normalization constant. The resulting pdfs are reported in Fig. S25.
